# Childhood maltreatment and risk of endocrine diseases: an exploration of mediating pathways using sequential mediation analysis

**DOI:** 10.1186/s12916-024-03271-9

**Published:** 2024-02-08

**Authors:** Shu Wen, Jianwei Zhu, Xin Han, Yuchen Li, Haowen Liu, Huazhen Yang, Can Hou, Shishi Xu, Junren Wang, Yao Hu, Yuanyuan Qu, Di Liu, Thor Aspelund, Fang Fang, Unnur A. Valdimarsdóttir, Huan Song

**Affiliations:** 1 Mental Health Center and West China Biomedical Big Data Center West China Hospital, Sichuan University, Guo Xue Lane 37, Chengdu, China; 2 Med-X Center for Informatics, Sichuan University, Chengdu, China; 3 Department of Critical Care Medicine, West China Hospital, Sichuan University, Chengdu, China; 4 Department of Orthopedic Surgery, West China Hospital, Sichuan University, Chengdu, China; 5 Mental Health Center, West China Hospital, Sichuan University, Chengdu, China; 6 Division of Endocrinology & Metabolism, West China Hospital, Sichuan University, Chengdu, China; 7 Sichuan University - Pittsburgh Institute, Sichuan University, Chengdu, China; 8 Center of Public Health Sciences, Faculty of Medicine, University of Iceland, Reykjavík, Iceland; 9 Institute of Environmental Medicine, Karolinska Institutet, Stockholm, Sweden; 10 Department of Epidemiology, Harvard T H Chan School of Public Health, Boston, MA USA

**Keywords:** Childhood maltreatment, Endocrine diseases, Sequential mediation analysis, Psychological adversities

## Abstract

**Background:**

Adverse childhood experiences (ACEs), including childhood maltreatment, have been linked with increased risk of diabetes and obesity during adulthood. A comprehensive assessment on the associations between childhood maltreatment and all major endocrine diseases, as well as the relative importance of different proposed mechanistic pathways on these associations, is currently lacking.

**Methods:**

Based on the UK Biobank, we constructed a cohort including 151,659 participants with self-reported data on childhood maltreatment who were 30 years of age or older on/after January 1, 1985. All participants were followed from the index date (i.e., January 1, 1985, or their 30th birthday, whichever came later) until the first diagnosis of any or specific (12 individual diagnoses and 9 subtypes) endocrine diseases, death, or the end of follow-up (December 31, 2019), whichever occurred first. We used Cox models to examine the association of childhood maltreatment, treated as continuous (i.e., the cumulative number of experienced childhood maltreatment), ordinal (i.e., 0, 1 and ≥ 2), or binary (< 2 and ≥ 2) variable, with any and specific endocrine diseases, adjusted for multiple covariates. We further examined the risk of having multiple endocrine diseases using Linear or Logistic Regression models. Then, sequential mediation analyses were performed to assess the contribution of four possible mechanisms (i.e., suboptimal socioeconomic status (SES), psychological adversities, unfavorable lifestyle, and biological alterations) on the observed associations.

**Results:**

During an average follow-up of 30.8 years, 20,885 participants received a diagnosis of endocrine diseases. We observed an association between the cumulative number of experienced childhood maltreatment and increased risk of being diagnosed with any endocrine disease (adjusted hazard ratio (HR) = 1.10, 95% confidence interval 1.09–1.12). The HR was 1.26 (1.22–1.30) when comparing individuals ≥ 2 with those with < 2 experienced childhood maltreatment. We further noted the most pronounced associations for type 2 diabetes (1.40 (1.33–1.48)) and hypothalamic–pituitary–adrenal (HPA)-axis-related endocrine diseases (1.38 (1.17–1.62)), and the association was stronger for having multiple endocrine diseases, compared to having one (odds ratio (95% CI) = 1.24 (1.19–1.30), 1.35 (1.27–1.44), and 1.52 (1.52–1.53) for 1, 2, and ≥ 3, respectively). Sequential mediation analyses showed that the association between childhood maltreatment and endocrine diseases was consistently and most distinctly mediated by psychological adversities (15.38 ~ 44.97%), while unfavorable lifestyle (10.86 ~ 25.32%) was additionally noted for type 2 diabetes whereas suboptimal SES (14.42 ~ 39.33%) for HPA-axis-related endocrine diseases.

**Conclusions:**

Our study demonstrates that adverse psychological sequel of childhood maltreatment constitutes the main pathway to multiple endocrine diseases, particularly type 2 diabetes and HPA-axis-related endocrine diseases. Therefore, increased access to evidence-based mental health services may also be pivotal in reducing the risk of endocrine diseases among childhood maltreatment-exposed individuals.

**Supplementary Information:**

The online version contains supplementary material available at 10.1186/s12916-024-03271-9.

## Background

Adverse childhood experiences (ACEs), including childhood maltreatment, encapsulate a broad spectrum of traumatic and distressing events that occur during childhood, mainly including direct maltreatment and neglect, physical and sexual abuse, violence, and household dysfunction [1]. Despite some variation in findings, numerous studies have reported a considerably high prevalence of ACEs (20–72%) among individuals with diverse demographic and socioeconomic characteristics [2–4]. Furthermore, besides the well-documented impacts on psychopathology during this critical period of development, growing evidence demonstrate profound and long-lasting influence of ACEs on a wide spectrum of somatic adversities, such as respiratory diseases, cardiovascular disease, cancer, and premature mortality [4–6].

The incidence of many endocrine diseases, i.e. disturbances of multiple hormone-producing cells and organs throughout the body, particularly diabetes and obesity, have been increasing in virtually all regions of the world [7]. A recent meta-analysis of 49 observational studies demonstrated a positive association between ACEs and diabetes, yielding an odds ratio of 1.22 for any ACEs and 1.27 for neglect [8]. Similarly, ACEs have been consistently associated with increased odds of childhood [9] and adult [10] obesity. However, despite evidence from human and animal studies supporting a wide range of endocrine dysfunctions which possibly involve all endocrine-related axes after ACEs [11–14], to date, there is no comprehensive evaluation of the association of ACEs with multiple endocrine diseases. Also, existing data are mainly derived from cross-sectional studies with limited sample size [10, 15, 16] and little focus on mediation factors [8, 16], which precludes the application of these data for causal inference or further mechanistic exploration.

Proposed explanations underlying the links between ACEs and somatic adversities in later life have been summarized and introduced in prior reviews published by the US Centers for Disease Control and Prevention [17], primarily including behavioral impairment (e.g., impaired coping strategies and unfavorable lifestyle) [18, 19], and socioeconomic deprivation (e.g., lower educational attainment and income) [19–21]. Such a conceptual framework has been widely used to elucidate the associations of ACEs with cardiovascular diseases [6] and psychiatric disorders [22]. Using prospective data from a US cohort, one recent study indicated the possible mediating role of metabolic syndrome on the association of ACEs with self-reported type 2 diabetes [23]. Another prospective cohort of British civil servants also demonstrated depression and cardiometabolic dysregulations might be the pathways linking ACEs and diabetes [24]. In addition, elevated susceptibility to psychiatric disorders as well as the dysregulation of hypothalamic–pituitary–adrenal (HPA) axis, a vital biological pathway that mediates the effects of stressors by influencing numerous physiological processes [25, 26], have also been observed among the ACE-exposed population [5]. Other possible biological alternations indicated in experimental studies include epigenetic changes, disruption of central neural networks, chronic activation of inflammatory pathways, and immune dysfunction [25, 27–29]. Nevertheless, as most of these studies focus on a specific type [10, 15, 23] or a small set [19, 23] of mediators, the attributable proportions of these mechanistic pathways, and consequently their importance for disease intervention, remain rather unexplored.

Leveraging data from the UK Biobank, which provides wealth information on childhood maltreatment, socioeconomic status (SES), psychological factors, lifestyle, biological biomarkers, as well as longitudinal and complete disease-related outcomes, we conducted a cohort study to assess the association between childhood maltreatment and multiple endocrine diseases during adulthood, with a particular focus on specific diagnoses (e.g., diabetes) and subtypes of endocrine diseases (e.g., HPA-axis-related endocrine diseases) to shed light on potential pathways. Furthermore, we focused on elucidating the relative importance of four specific mechanistic pathways (i.e., suboptimal SES, psychological adversities, unfavorable lifestyles, and biological alterations) using sequential causal mediation analysis, which is a newly developed method for weighting the contribution of multiple causally related mediators after accommodating exposure-mediator interactions and various assumptions about the causal ordering of these mediators [30].

## Methods

### Data source and study design

The study was based on the UK Biobank, which enrolled 502,507 participants aged 40–69 years across England, Scotland, and Wales between 2006 and 2010. The UK Biobank is not representative of the general population in the UK, as it recruited only 5.5% of the invited population and the participants were predominately white (94.6%) [31]. At recruitment, data on sociodemographic characteristics, lifestyles and psychosocial factors, medical and family history, as well as physical measures were collected using touchscreen questionnaire [32]. Health-related outcomes including medical consequences and survival status were obtained through periodical linkages to multiple national datasets [33]. Information on inpatient hospital care was derived from Hospital Episode Statistics for England, Scottish Morbidity Record, and Patient Episode Database for Wales, covering almost all UK Biobank participants since 1997 [33]. Primary care data was obtained from numerous general practice systems and covers approximately 45% of the UK Biobank participants [33].

In the present study, we first identified 153,634 participants that responded to all five questions about ACEs (mainly childhood maltreatment) in the touchscreen questionnaire (see comparison on sociodemographic characteristics of participants with and without such data in Additional file 1: Table S1) and excluded 41 participants who withdrew their informed consent afterwards. Then, as data from primary care has been available since 1985 (most UK Biobank participants were above 58 at recruitment), we focused on endocrine diseases diagnosed at age 30 or after. Accordingly, after exclusion of 1934 participants with a history of endocrine diseases, we constructed a cohort including 151,659 participants who were 30 years of age or older on/after January 1, 1985. All participants were followed from the index date (i.e., January 1, 1985, or their 30th birthday, whichever came later), until the first diagnosis of any or specific endocrine diseases (12 individual diagnoses and 9 subtypes), death, or the end of follow-up (December 31, 2019), whichever occurred first.

#### Assessment of childhood maltreatment

In 2016, childhood maltreatment was assessed by five items (i.e., physical abuse, emotional abuse, sexual abuse, sexual neglect, and physical neglect) in an online mental health questionnaire survey, using the Childhood and Trauma Screener [34] (See Additional file 1: Table S2 for details). For instance, the online question for physical abuse is “When I was growing up: People in my family hit me so hard that it left me with bruises or marks”. Each item was dichotomized (yes or no) using cut-off points reported previously [35] (Additional file 1: Table S2) and the five items were then summed to generate the cumulative number of childhood maltreatment.

#### Ascertainment of endocrine diseases

We ascertained endocrine diseases based on diagnoses derived from the self-reported questionnaire, and linked data from primary care and hospital inpatient registers, using the International Classification of Diseases 10th edition (ICD-10: E00-E34). The mapping of the diagnoses from different data sources was performed through an expert peer review and consensus process, providing reliable information on the diagnosis date of the first occurrence of a set of specific health outcomes [36]. Besides considering all endocrine diseases as one outcome, we additionally identified 12 individual diagnoses of endocrine diseases, and 9 subtypes of endocrine diseases according to the involved gland (i.e., thyroid, pancreatic, parathyroid, hypothalamic-pituitary, adrenal, genital glands) or axis (i.e., HPA, hypothalamic-pituitary-thyroid [HPT] and hypothalamic-pituitary–gonadal [HPG] axis). All ICD-10 codes used for endocrine disease identification could be found in Additional file 1: Table S3.

#### Covariates and mediators

Given the well-established influences of demographic factors, childhood SES condition, and genetic susceptibility to disease on the studied associations [37, 38], we obtained information on multiple covariates, including sociodemographic characteristics (i.e., sex, birth year, ethnicity, and country of birth), childhood environment (i.e., number of siblings, maternal smoking, and being breastfed), and family history of diabetes, from the baseline survey (see Additional file 1: Table S2 for details).

Data on candidate mediators was collected at baseline. To test the importance of the four hypothesized pathways, we categorized them in four categories (i.e., SES, psychological factors, lifestyle factors, and biological biomarkers). Specifically, the SES category included Townsend Deprivation Index (TDI), educational level, household income, and employment status. Psychological factors included items related to self-rated mental problems [6] and social support (i.e., ability to confide in others, frequency of friend/family visits, and leisure/social activities) according to the baseline survey. Lifestyle factors indicated status of physical activity, smoking, alcohol, sleep pattern, diet, body mass index (BMI), systolic pressure, and diastolic pressure. In addition, as biomarkers of inflammation, nutrition, lipid, and glucose have been considered important to the associations between ACEs and the occurrence of diseases [27], we included C-reactive protein, total protein, Ca, Vitamin D, cholesterol, lipoprotein A, high-density lipoprotein (HDL), low-density lipoprotein (LDL), apolipoprotein A, apolipoprotein B, triglycerides, glucose, and glycosylated hemoglobin in the category of biomarkers.

For all variables, the answers “do not know” or “prefer not to answer” were considered as unknown. Details of the included mediators are listed in Additional file 1: Table S2 and Additional file 2: Supplementary method [6, 39–43].

### Statistical analysis

To assess the association of childhood maltreatment, treated as continuous (i.e., the cumulative number of experienced childhood maltreatment), ordinal (i.e., 0, 1, and ≥ 2), or binary (< 2 and ≥ 2) variable, with any endocrine diseases diagnosed after age of 30, we applied Cox models, partly (models 1–3) or fully (model 4) adjusted for all covariates, calculating hazard ratios (HRs) with their 95% confidence intervals (CIs). We then performed separate analyses for different types of experienced childhood maltreatment, as well as for 12 individual diagnoses and 9 subtypes of endocrine diseases. Further, we evaluated the association of childhood maltreatment (as a binary variable: < 2 and ≥ 2) with multiple endocrine diseases (i.e., the number of any endocrine disease and the number of HPA-axis-related endocrine diseases), using Linear or Logistic Regression models.

**Fig. 1:**
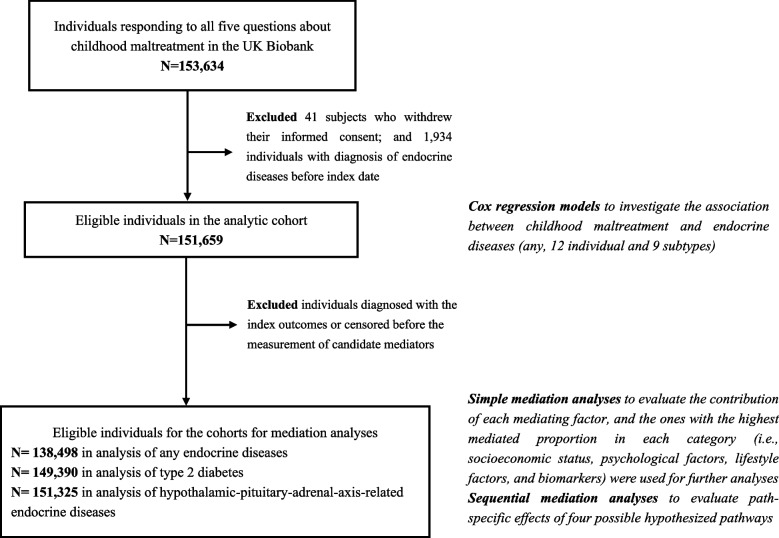
Study design

Next, we performed the sequential mediation analyses to assess the contribution of four possible mechanisms (i.e., suboptimal SES, psychological adversities, unfavorable lifestyle, and biological alterations) to the associations of childhood maltreatment (as a binary variable: < 2 and ≥ 2) with any endocrine disease, type 2 diabetes, and HPA-axis-related endocrine diseases, in three steps. First, to ensure a temporal order between childhood maltreatment, mediators, and endocrine diseases, we restricted the analyses to participants with no censoring event (i.e., diagnosis of endocrine diseases, death, or loss of follow-up) before the recruitment to UK Biobank. The comparison between the new cohort (*n* = 138,498 for any endocrine disease cohort, Fig. 1) and the entire cohort of the present study showed a comparable distribution of baseline characteristics (Additional file 1: Table S4). Second, as we included several mediators in each hypothesized pathway, we conducted a simple mediation analysis to evaluate the contribution of each mediator. Specifically, using “CMAverse” package based on the counterfactual g-formula approach [44], the mediated proportion of each mediator was estimated by partitioning the total effect of exposure-outcome association into direct effect and indirect effect with causal mediation framework [45]. Parametric bootstrapping (100 times) was used to calculate 95% CIs and *P* value. We then chose the mediators with the greatest mediated proportion in each category for further analyses. Third, in the sequential mediation analyses, a multi-mediator model [30] was used to measure the mediation proportion of different pathways through assessing the path-specific effects [46], using Cox models adjusted for abovementioned covariates. Namely, by defining the selected variables for SES, psychological factors, lifestyle factors, and biomarkers as M1, M2, M3, and M4, respectively, we calculated the direct effect, the effect of M1-mediated pathways, the effect of M2-mediated pathways, the effect of M3-mediated pathways, and the effect of M4-mediated pathways, as components of the total effect. This calculation was done for all possible temporal orders of the suggested attributable mediators (e.g., M1 → M2 → M3 → M4 and M1 → M2 → M4 → M3, see proposed causal diagram in Fig. 3A and B). Particularly, as the multi-mediator models do not allow missing values, individuals with missing data of included mediators were excluded from the analysis. We then summarized the mediated proportions of each hypothesized pathway by the range (lowest-highest) of point estimates derived from different sequential models. Detailed explanations of the mediation analyses are provided in Additional file 2: Supplementary method [30, 46–51].

In the sensitivity analyses, we applied a competing risk model to re-assess the studied associations, taking into account competing risk by death. To assess the soundness of the findings by the definition of childhood maltreatment exposure, we re-estimated the mediation proportions using an alternative grouping strategy (i.e., number of experienced childhood maltreatment ≥ 1 vs < 1), or after restricting the reference group to individuals without any childhood maltreatment experience (i.e., ≥ 2 *vs* 0). In addition, we assessed the influence of missingness in some mediators (0.13–14.02%, see Additional file 1: Table S5) to the obtained estimates by repeating the sequential mediation analyses after performing multiple imputation. All statistical analyses were conducted using R, version 4.0.2 (R Project for Statistical Computing). A two-sided *P* < 0.05 was considered statistically significant.

## Results

Among 151,659 participants included in full cohort for the association analyses, the mean age at cohort entry was 34.6 years (standard deviation (SD) = 4.85) and 43.93% of them were male (Table 1). While there were few discrepancies regarding demographic characteristics between groups with a different exposure level of childhood maltreatment (0, 1, and ≥ 2), individuals exposed to a higher level of childhood maltreatment exposure were more likely to be women (55.79% for *n* = 0 [i.e., no childhood maltreatment] *vs* 53.59% for *n* = 1 and 59.78% for ≥ 2 group), have ≥ 3 siblings (21.94% *vs* 28.29% and 35.06%), have a family history of diabetes (20.35% *vs* 21.17% and 23.74%), and have reported maternal smoking (22.64% *vs* 25.43% and 31.06%), but less likely to have been breastfed (60.69% *vs* 58.56% and 54.94%) (Table 1).

**Table 1:** Baseline characteristics of the study population

	Different levels of experienced childhood maltreatment			Overall (*N* = 151,659)
	0 *N* = 81,372 (53.65%)	1 *N* = 38,728 (25.54%)	≥ 2 *N* = 31,559 (20.81%)	
**Birth year**				
Mean (SD)	1950 (7.66)	1950 (7.74)	1950 (7.77)	1950 (7.72)
**Age at the index date, year**				
Mean (SD)	34.7 (4.83)	34.8 (4.93)	34.1 (4.74)	34.6 (4.85)
**Age at recruitment, year**				
Mean (SD)	56.6 (7.67)	56.7 (7.75)	55.6 (7.78)	56.5 (7.73)
**Follow-up time, year**				
Mean (SD)	31.0 (6.37)	30.9 (6.48)	30.1 (6.94)	30.8 (6.53)
**Sex, No. (%)**				
Female	45,396 (55.79)	20,755 (53.59)	18,865 (59.78)	85,016 (56.06)
Male	35,976 (44.21)	17,973 (46.41)	12,694 (40.22)	66,643 (43.93)
**Place of birth, No. (%)**				
England	65,970 (81.07)	30,858 (79.68)	24,893 (78.88)	121,721 (80.26)
Wales	3614 (4.44)	1524 (3.94)	1122 (3.56)	6260 (4.13)
Scotland	6342 (7.79)	3061 (7.90)	2316 (7.34)	11,719 (7.73)
Elsewhere	5394 (6.63)	3258 (8.41)	3196 (10.13)	11,848 (7.81)
Unknown	52 (0.06)	27 (0.07)	32 (0.10)	111 (0.07)
**Ethnicity, No. (%)**				
White	76,004 (93.40)	35,417 (91.45)	27,872 (88.3)	139,293 (91.85)
Others	5134 (6.31)	3172 (8.19)	3555 (11.3)	11,861 (7.82)
Unknown	234 (0.29)	139 (0.36)	132 (0.4)	505 (0.33)
**Number of siblings, No. (%)**				
0	11,420 (14.03)	4547 (11.74)	3165 (10.03)	19,132 (12.62)
1	30,888 (37.96)	13,054 (33.71)	9189 (29.12)	53,131 (35.03)
2	21,027 (25.84)	10,085 (26.04)	8020 (25.41)	39,132 (25.80)
≥ 3	17,856 (21.94)	10,957 (28.29)	11,064 (35.06)	39,877 (26.29)
Unknown	181 (0.22)	85 (0.22)	121 (0.38)	387 (0.26)
**Maternal smoking, No. (%)**				
No	53,251 (65.44)	23,957 (61.86)	17,384 (55.08)	94,592 (62.37)
Yes	18,426 (22.64)	9850 (25.43)	9802 (31.06)	38,078 (25.11)
Unknown	9695 (11.91)	4921 (12.71)	4373 (13.86)	18,989 (12.52)
**Being breastfed, No. (%)**				
No	16,734 (20.56)	8017 (20.70)	7222 (22.88)	31,973 (21.08)
Yes	49,384 (60.69)	22,678 (58.56)	17,338 (54.94)	89,400 (58.95)
Unknown	15,254 (18.75)	8033 (20.74)	6999 (22.18)	30,286 (19.97)
**Family history of diabetes, No. (%)**				
No	64,813 (79.65)	30,528 (78.83)	24,068 (76.26)	119,409 (78.74)
Yes	16,559 (20.35)	8200 (21.17)	7491 (23.74)	32,250 (21.26)

*Abbreviation*: *SD* standard deviation

During an average follow-up of 30.8 years (SD = 6.53), we identified 20,885 individuals who developed endocrine diseases. The number of cases was 10,394, 5312, and 5179 among individuals with 0, 1, or ≥ 2 childhood maltreatment, corresponding to a crude incidence rate (IR) of 4.12, 4.44, and 5.45 per 1000 person-years, respectively. As shown in Table 2, we found a positive association between the cumulative number of experienced childhood maltreatment and any endocrine disease (birth year- and sex-adjusted HR (model 1) = 1.12 (95% CI 1.11–1.13); and fully adjusted HR (model 4) = 1.10 (95% CI 1.09–1.12)). Compared with individuals without childhood maltreatment, the HR of developing any endocrine diseases was 1.07 (95% CI 1.04–1.11) and 1.29 (95% CI 1.25–1.33) for individuals exposed to 1 or ≥ 2 childhood maltreatment experiences, respectively, according to the fully adjusted model. The HR was 1.26 (95% CI 1.22–1.30) when comparing individuals with ≥ 2 to those with < 2 childhood maltreatment experiences. Also, we observed comparable estimates for different types of childhood maltreatment, with fully adjusted HRs ranging from 1.17 (95% CI 1.14–1.21) for emotional neglect to 1.23 (95% CI 1.19–1.27) for physical neglect (Table 2).

**Table 2:** Hazard ratios (HRs) with 95% confidence intervals (CIs) for the associations between childhood maltreatment and any endocrine disease

	Number of cases (incidence rate, per 1000 person years)	Model 1^a^	Model 2^b^	Model 3^c^	Model 4^d^
		HR (95% CI)	HR (95% CI)	HR (95% CI)	HR (95% CI)
Cumulative number of experienced childhood maltreatment (as continuous variable)					
	20,885 (4.47)	1.12 (1.11–1.13)	1.12 (1.11–1.13)	1.11 (1.09–1.12)	1.10 (1.09–1.12)
Different levels of experienced childhood maltreatment (as ordinal variable)					
0	10,394 (4.12)	Ref	Ref	Ref	Ref
1	5312 (4.44)	1.09 (1.05–1.12)	1.08 (1.05–1.12)	1.07 (1.04–1.11)	1.07 (1.04–1.11)
≥ 2	5179 (5.45)	1.34 (1.30–1.39)	1.34 (1.29–1.38)	1.30 (1.26–1.34)	1.29 (1.25–1.33)
Different levels of experienced childhood maltreatment (as binary variable)					
< 2	15,706 (4.22)	Ref	Ref	Ref	Ref
≥ 2	5179 (5.45)	1.31 (1.27–1.35)	1.30 (1.26–1.34)	1.27 (1.23–1.31)	1.26 (1.22–1.30)
Different types of experienced childhood maltreatment					
Physical abuse					
No	16,585 (4.34)	Ref	Ref	Ref	Ref
Yes	4300 (5.02)	1.23 (1.19–1.27)	1.23 (1.19–1.27)	1.20 (1.16–1.24)	1.19 (1.15–1.23)
Emotional abuse					
No	17,185 (4.32)	Ref	Ref	Ref	Ref
Yes	3700 (5.32)	1.25 (1.21–1.30)	1.25 (1.20–1.29)	1.22 (1.18–1.27)	1.21 (1.17–1.26)
Sexual abuse					
No	18,649 (4.36)	Ref	Ref	Ref	Ref
Yes	2236 (5.61)	1.25 (1.19–1.30)	1.24 (1.19–1.30)	1.22 (1.17–1.27)	1.21 (1.16–1.26)
Emotional neglect					
No	15,654 (4.28)	Ref	Ref	Ref	Ref
Yes	5231 (5.13)	1.21 (1.17–1.25)	1.21 (1.17–1.24)	1.18 (1.14–1.21)	1.17 (1.14–1.21)
Physical neglect					
No	16,661 (4.25)	Ref	Ref	Ref	Ref
Yes	4224 (5.57)	1.26 (1.22–1.30)	1.26 (1.21–1.30)	1.23 (1.19–1.27)	1.23 (1.19–1.27)

HRs with 95% CIs were derived from Cox models:

^a^The estimates were adjusted for sex and birth year

^b^The estimates were adjusted for sex, birth year, ethnicity, and country of birth

^c^The estimates were adjusted for sex, birth year, ethnicity, country of birth, number of siblings, maternal smoking, and being breastfed

^d^The estimates were adjusted for sex, birth year, ethnicity, country of birth, number of siblings, maternal smoking, being breastfed, and family history of diabetes

**Fig. 2:**
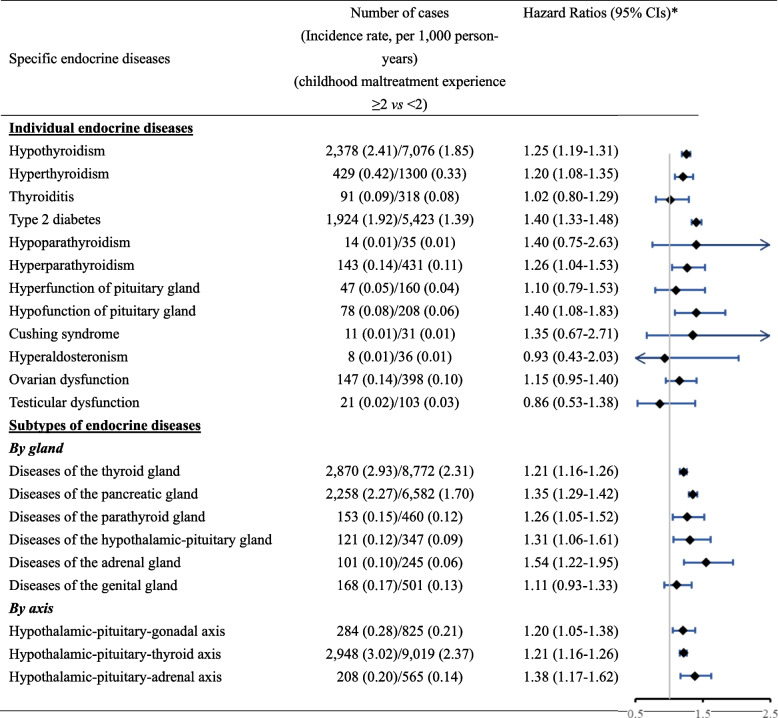
Hazard ratios (HRs) with 95% confidence intervals (CIs) for the association between childhood maltreatment (≥ 2 *vs* < 2) and specific (individual or subtypes) endocrine diseases. * Cox models were adjusted for sex, birth year, ethnicity, country of birth, number of siblings, maternal smoking, being breastfed, and family history of diabetes

Among the 12 studied individual diagnoses of endocrine diseases, five (i.e., hypothyroidism, hyperthyroidism, type 2 diabetes, hyperparathyroidism, and hypofunction of teh pituitary gland) showed significant associations with prior childhood maltreatment exposure, as either ordinal (Additional file 1: Table S6) or binary (Fig. 2) variable, with the most pronounced associations noted for type 2 diabetes. In the analyses of subtypes, we observed excess risks of developing endocrine diseases in most studied glands, particularly adrenal (1.54 (95% CI 1.22–1.95)), pancreatic (1.35 (95% CI 1.29–1.42)), and hypothalamic-pituitary (1.31 (95% CI 1.06–1.61)) glands, among childhood maltreatment-exposed individuals (Fig. 2). In the analysis of the involved neuroendocrine axis, the highest HR was found for HPA-axis-related endocrine diseases (1.38 (95% CI 1.17–1.62)) (Fig. 2). The association was stronger for having multiple endocrine diseases, compared to having one disease only (odds ratio = 1.24 (95% CI 1.19–1.30), 1.35 (95% CI 1.27–1.44), 1.52 (95% CI 1.52–1.53) for having 1, 2, ≥ 3 endocrine diseases, respectively) (Additional file 1: Table S7). Similar results were found for the number of HPA-axis-related endocrine diseases.

**Fig. 3:**
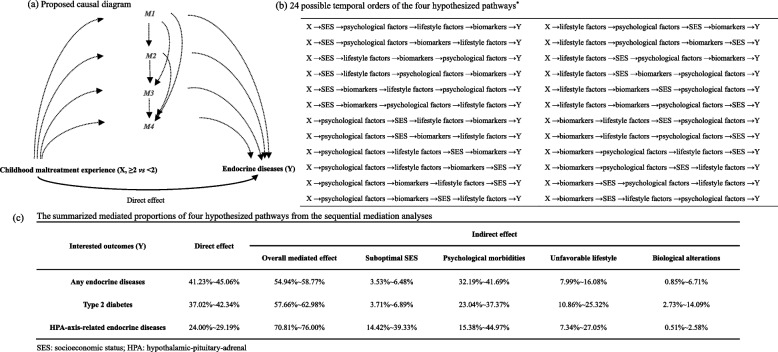
The proposed casual diagram with possible temporal orders and the summarized mediated proportion of four hypothesized pathways from the sequential mediation analyses. * The selected mediators for each hypothesized pathway were Townsend Deprivation Index for suboptimal SES, self-rated mental problem for psychological morbidities, body mass index for unfavorable lifestyle, and high-density lipoprotein for biological alternations in the cohorts for mediation analyses of any endocrine diseases and type 2 diabetes, as well as employment, self-rated mental problem, alcohol and triglycerides in the cohort for mediation analyses of HPA-axis-related endocrine diseases, according to the results of simple mediation analyses

We performed the mediation analyses for any endocrine disease, type 2 diabetes, and HPA-related endocrine diseases. First, we demonstrated that the observed associations remained valid in the cohorts constructed for mediation analyses (i.e., HR = 1.30 (95% CI 1.24–1.37), 1.38 (95% CI 1.29–1.47), and 1.44 (95% CI 1.17–1.79) for any endocrine disease, type 2 diabetes, and HPA-related endocrine diseases, respectively (Additional file 1: Table S8)). The results of simple mediation analyses for all candidate mediators are shown in Additional file 1: Table S9. For any endocrine disease, we accordingly selected the mediator with the greatest mediated proportion in each hypothesized pathway (i.e., TDI (mediated proportion = 9.14%) for SES, self-rated mental problem (mediated proportion = 20.06%) for psychological factors, BMI (mediated proportion = 21.45%) for lifestyle factors, and HDL (mediated proportion = 11.87%) for biomarkers) in the sequential mediation analysis. Then, sequential mediation analyses on 24 possible temporal orders indicated a direct effect of 41.23 ~ 45.06% between childhood maltreatment and any endocrine disease, as well an indirect effect of 32.19 ~ 41.69%, 7.99 ~ 16.08%, 3.53 ~ 6.48%, and 0.85 ~ 6.71% mediated by psychological adversities, unfavorable lifestyle, suboptimal SES, and biological alterations, respectively (Fig. 3C). With the identical analytic process, we found smaller proportions of direct effect for type 2 diabetes (37.02 ~ 42.35%) and HPA-axis-related endocrine diseases (24.00 ~ 29.19%) (Additional file 1: Tables S10–S12 and Fig. 3C). In addition, although psychological adversities consistently showed the greatest mediated proportion, a notable mediating role of unfavorable lifestyle (10.86 ~ 25.32%) was observed for type 2 diabetes, and suboptimal SES (14.42 ~ 39.33%) was found for HPA-axis-related endocrine diseases (Fig. 3C).

The application of competing risk models resulted in largely identical estimates in the association analyses (Additional file 1: Table S13). In addition, neither using an alternative grouping strategy (i.e., childhood maltreatment ≥ 1 vs < 1) nor considering merely individuals without any childhood maltreatment as the reference group (i.e., ≥ 2 vs 0) changed the findings of mediation analyses (Additional file 1: Table S14). Also, in the sensitivity analysis where multiple imputation was applied to deal with the missing values of selected mediators, largely similar results were obtained (Additional file 1: Table S14).

## Discussion

In the community-based cohort study of UK Biobank, we found that individuals exposed to childhood maltreatment were at increased risk of developing any and specific (e.g., type 2 diabetes and HPA-axis-related) endocrine diseases in adulthood. The sequential mediation analyses suggested a considerable contribution of studied hypothesized pathways on the observed associations (54.94–76.00%), with the most notable mediating effect by psychological adversities. The importance of suboptimal SES was additionally noted for HPA-axis-related endocrine diseases after exposure to childhood maltreatment, while unfavorable lifestyle was considered critical for the link from childhood maltreatment to type 2 diabetes. These findings shed light on potential targets for interventions, such as early psychological interventions, to reduce the subsequent risk of endocrine diseases in childhood maltreatment-exposed population.

Our findings of an association between childhood maltreatment and subsequent endocrine diseases are consistent with previous studies, most of which, however, focused on diabetes [15]. For instance, the results of two meta-analyses including a total of 20 observational studies concluded that individuals exposed to sexual abuse had 39% [52] and 25% [8] increased risk of diabetes, respectively, later in life. Our study extends the body of existing knowledge by demonstrating an association of childhood maltreatment with any and multiple endocrine diseases, in line with previous research demonstrating the effects of ACEs on various endocrine axes, such as the initial hyperactivity and the subsequent hypoactivity of HPA axis [11], modified basal HPT functions [12], faster sexual maturation [13], and the alternations of hormone secretion [53]. Moreover, the pronounced risk elevation observed for HPA-axis-related endocrine diseases [54] and the high mediated proportion by psychological adversities corroborate the prevailing notion that ACEs can be “biologically embedded” through its persistent or chronic impact on psychopathology and the accompanied HPA-axis dysfunction [27]. Indeed, using disease trajectory analyses, individuals with psychiatric disorders, such as depression [55], showed increased risks of various endocrine diseases. Also, human and animal studies have indicated that progressive dysfunction of HPA axis can lead to insulin resistance and visceral obesity [56] and thereby increase the possibility of developing diabetes, as well as endocrine diseases in all HPA-involved glands (e.g., Cushing syndrome) [57]. Nevertheless, prior efforts are scarce in exploring the association between childhood maltreatment and other endocrine diseases, apart from limited evidence on thyroid diseases or ovarian dysfunction in some specific populations [53, 58]. Our findings, however, corroborated with several studies reporting signals of hormone abnormalities, such as reduced thyroid-hormone T3 [59] and symptoms of polycystic ovary syndrome [58], among individuals exposed to childhood maltreatment.

The underlying mechanisms through which ACEs, including childhood maltreatment, can contribute to the pathogenesis of various somatic diseases, such as endocrine diseases, remain inconclusive. According to the existing literature, we placed our focus of mediation analyses on four proposed mechanistic pathways, i.e., psychological adversities, suboptimal SES, unfavorable lifestyle, and biological alterations. Psychological stress and corresponding lifelong mental morbidity is a well-documented consequence of ACEs [60], which could subsequently affect functions of HPA axis through changes of neuroendocrine cells, brain structures (e.g., amygdala and hippocampus) [61], and epigenetic factors [62]. The role of chronic inflammation and immune suppression, as a result of HPA axis dysfunction and insufficient glucocorticoid signaling, has been well-established in the pathogenesis of type 2 diabetes and endocrine-associated complications [63, 64] through crosstalk between inflammatory system and thyroid or reproductive function [64, 65]. Moreover, a recent study also highlighted the DNA methylation as the novel mechanism linking HPA axis and immune dysregulation [66]. Similarly, SES disadvantages (e.g., lower educational attainment, deprivation, and unemployment status) have consistently been reported among ACEs-exposed individuals [19], which could increase susceptibility to diseases via reduced healthcare seeking and limited access to the healthcare system [18]. Furthermore, as childhood is a sensitive period of neurodevelopment, ACEs may affect behavioral regulations [67] and vulnerabilities for addiction to, e.g., cannabis [67] and alcohol [4] to cope with the emotional pain following childhood maltreatment, while these addictions may contribute to subsequent health hazards, including to the development of endocrine disease. Finally, as biological changes, including chronic inflammation and undermined immune suppression, and other imbalances (e.g., autonomic imbalances), are likely induced by childhood maltreatment (and all abovementioned mediators), it is plausible that the status of biomarkers, particularly inflammatory biomarkers [27, 29, 62], could reflect the extent to which childhood maltreatment influence the pathophysiology towards endocrine diseases.

However, while relatively abundant attempts on clarifying the potential mediating effect of different mechanistic pathway, studies are currently lacking in understanding the joint effects as well as in quantifying the mediated proportions of these pathways. Therefore, our study filled this knowledge gap by demonstrating a considerable proportion (54.96–76.00%) of indirect effect on the associations between childhood maltreatment and endocrine diseases, particularly type 2 diabetes and HPA-axis-related endocrine diseases. In addition to the greatest mediating proportion observed for psychological adversities, as expected, our results also demonstrate a mediating role of unfavorable lifestyle for type 2 diabetes. This is in line with previous reports and further underscores the importance of behavior modifications for diabetes prevention in childhood maltreatment-exposed individuals. Likewise, the notable mediating effect of suboptimal SES on the link between childhood maltreatment and HPA-axis-related endocrine diseases is plausible, as lower SES in adulthood could act as an adverse stimulus on the stress response system. Individuals with lower SES might also have less social support and material resources to handle stressful events [68]. Finally, because the biological characteristics were measured only once and at an average age of 56 years in the UK Biobank, the smaller mediated proportion of biological pathways observed in the present study should not preclude the importance of examining biomarkers in future studies.

The strengths of the present study include the large study population and longitudinal data derived from the UK Biobank. For instance, the diagnoses of endocrine diseases were prospectively obtained through data linkage with nationwide healthcare registers, which minimizes the possibility of information bias. Also, with most candidate mediators measured at recruitment, the clear temporal order between childhood maltreatment, mediating factors, and the diagnosis of endocrine diseases provides a solid basis for the mediation analyses. In addition, in contrast to the traditional mediation analyses requiring strong and unverifiable assumptions, the application of sequential mediation analyses enables the measure of path-specific mediated proportion accounting for different sequential assumptions for all involved pathways. Consequently, the range of estimates obtained under 24 possible temporal orders could, from a global perspective, be used to elucidate the relative importance of several involved mediators. This analytic strategy has been successfully applied in projects with similar objectives, e.g., evaluating mediating factors important for the association between childhood maltreatment and cardiovascular diseases [6].

There are also limitations in the study. First, the status of childhood maltreatment was defined based on five self-rated questions collected many years after actual exposure (e.g., in 2016). Therefore, the concern of misclassification due to recall bias may to some extent distort the observed associations. However, several prior studies have demonstrated a certain consistency between retrospective and prospective measures of childhood maltreatment, although the use of retrospectively reported childhood maltreatment poses the risk of underestimating the impact of ACEs on objectively measured life adversities [69, 70]. Additionally, apart from childhood maltreatment, other types of ACEs were not measured or addressed in the present study, such as those ones related to childhood household dysfunctions (e.g., childhood household mental illness and childhood parental death) that have been linked with adverse health consequences [8]. Second, despite the enrichment of available data on candidate mediators in the UK Biobank, these factors were measured only once at recruitment which could not accurately reflect the long-term status. Exposure (other than childhood maltreatment) might also have influenced these mediators and cannot fully considered. Third, our mediation analyses were performed for four pre-defined pathways. Other mechanistic pathways might have also contributed to the observed association and need to be investigated in future explorations. Last, the UK Biobank sample is not representative of the general UK population, as the participants were more likely white, healthier, older, and less socioeconomically deprived than nonparticipants [31]. Also, we have noted a lower SES status for the participants who provided data on childhood maltreatment, compared to those without. The generalizability of our findings to the whole UK or other populations needs to be evaluated further.

## Conclusions

In conclusion, based on the community-based prospective cohort of UK Biobank, our study demonstrated the association between childhood maltreatment and multiple endocrine diseases is primarily driven by the mediating role of psychological adversities. The importance of suboptimal SES was additionally noted for HPA-axis-related endocrine diseases and of unfavorable lifestyle was noted for type 2 diabetes. These findings therefore carry an important message for prevention, namely that the targeted interventions (such as early psychological interventions) may reduce the downstream risk of endocrine diseases in the childhood maltreatment-exposed population.

### Supplementary Information

**Additional file 1: Table S1.** The distribution of sociodemographic factors of UK Biobank participants. **Table S2.** The definition of exposure, covariates and candidate mediators in the present study. **Table S3.** International Classification of Diseases 10th edition (ICD-10) codes used to ascertain endocrine diseases in this study. **Table S4.** Baseline characteristics of study population in the cohort for mediation analyses (*n*=138,498). **Table S5.** Distribution of candidate mediators among individuals with different levels of childhood maltreatment experience (≥ 2 vs <2) in the cohorts for mediation analyses. **Table S6.** Hazard ratios (HRs) with 95% confidence intervals (CIs) for the association between childhood maltreatment experience (=1 and ≥ 2 vs 0) and specific (individual diagnoses or subtypes) endocrine diseases. **Table S7.** The association of childhood maltreatment experience (≥2 vs <2) and the number of any or HPA-axis-related endocrine diseases. **Table S8.** Hazard ratios (HRs) with 95% confidence intervals (CIs) for the association between childhood maltreatment experience (≥2 vs <2) with any and specific (individual diagnoses or subtypes) endocrine diseases in the cohorts for mediation analyses. **Table S9.** Estimated mediated proportion of candidate mediators for the association of childhood maltreatment with any and specific endocrine diseases, using simple mediation analyses. **Table S10.** Estimated mediated proportion of selected mediators, by different temporal orders, for the association between childhood maltreatment and any endocrine diseases, using sequential mediation analyses. **Table S11.** Estimated mediated proportion of selected mediators, by different temporal orders, for the association between childhood maltreatment and type 2 diabetes, using sequential mediation analyses. **Table S12.** Estimated mediated proportion of selected mediators, by different temporal orders, for the association between childhood maltreatment and HPA-axis-related endocrine diseases, using sequential mediation analyses. **Table S13.** The competing risk of death in the relationship between childhood maltreatment and endocrine diseases. **Table S14.** Sensitivity analyses for the summarized mediated proportions of four hypothesized pathways from the sequential mediation analyses.

**Additional file 2.** Supplementary method.

## Data Availability

Data from UK Biobank are available per the researchers request (https://www.ukbiobank.ac.uk/enable-your-research/apply-for-access). This research was done using the UK Biobank Resource under Application 54803 (approved on October 29, 2019).

## Abbreviations

ACE: Adverse childhood experience
BMI: Body mass index
CI: Confidence interval
HDL: High-density lipoprotein
HPA: Hypothalamic-pituitary-adrenal
HPG: Hypothalamic-pituitary–gonadal
HPT: Hypothalamic-pituitary-thyroid
HR: Hazard ratio
ICD-10: International Classification of Diseases 10th edition
LDL: Low-density lipoprotein
SD: Standard deviation
SES: Socioeconomic status
TDI: Townsend Deprivation Index
