# Supplementary material for: Childhood maltreatment and risk of endocrine diseases: an exploration of mediating pathways using sequential mediation analysis
Source: BMC Med. 2024 Feb 8;22:59. doi: 10.1186/s12916-024-03271-9 (PMC10854183; doi:10.1186/s12916-024-03271-9)
Supplement: Supplementary file 1 — Additional file 1: Table S1. The distribution of sociodemographic factors of UK Biobank participants. Table S2. The definition of exposure, covariates and candidate mediators in the present study. Table S3. International Classification of Diseases 10th edition (ICD-10) codes used to ascertain endocrine diseases in this study. Table S4. Baseline characteristics of study population in the cohort for mediation analyses (n=138,498). Table S5. Distribution of candidate mediators among individuals with different levels of childhood maltreatment experience (≥ 2 vs <2) in the cohorts for mediation analyses. Table S6. Hazard ratios (HRs) with 95% confidence intervals (CIs) for the association between childhood maltreatment experience (=1 and ≥ 2 vs 0) and specific (individual diagnoses or subtypes) endocrine diseases. Table S7. The association of childhood maltreatment experience (≥2 vs <2) and the number of any or HPA-axis-related endocrine diseases. Table S8. Hazard ratios (HRs) with 95% confidence intervals (CIs) for the association between childhood maltreatment experience (≥2 vs <2) with any and specific (individual diagnoses or subtypes) endocrine diseases in the cohorts for mediation analyses. Table S9. Estimated mediated proportion of candidate mediators for the association of childhood maltreatment with any and specific endocrine diseases, using simple mediation analyses. Table S10. Estimated mediated proportion of selected mediators, by different temporal orders, for the association between childhood maltreatment and any endocrine diseases, using sequential mediation analyses. Table S11. Estimated mediated proportion of selected mediators, by different temporal orders, for the association between childhood maltreatment and type 2 diabetes, using sequential mediation analyses. Table S12. Estimated mediated proportion of selected mediators, by different temporal orders, for the association between childhood maltreatment and HPA-axis-related endocrine diseases, using [file 12916_2024_3271_MOESM1_ESM.docx]

**_Childhood maltreatment and risk of endocrine diseases: an exploration of mediating pathways using sequential mediation analysis_**

**Additional file 1**

Shu Wen; Jianwei Zhu; Xin Han; Yuchen Li; Haowen Liu; Huazhen Yang; Can Hou; Shishi Xu; Junren Wang; Yao Hu; Yuanyuan Qu; Di Liu; Thor Aspelund; Fang Fang; Unnur A Valdimarsdóttir; Huan Song

**Additional file 1**

**Contents**

[Table S1 The distribution of sociodemographic factors of UK Biobank participants 3](#_Toc156565447)

[Table S2 The definition of exposure, covariates and candidate mediators in the present study 5](#_Toc156565448)

[Table S3 International Classification of Diseases 10th edition (ICD-10) codes used to ascertain endocrine diseases in this study 10](#_Toc156565449)

[Table S4 Baseline characteristics of study population in the cohort for mediation analyses (n=138,498) 11](#_Toc156565450)

[Table S5 Distribution of candidate mediators among individuals with different levels of childhood maltreatment experience (≥ 2 *vs* <2) in the cohorts for mediation analyses 13](#_Toc156565451)

[Table S6 Hazard ratios (HRs) with 95% confidence intervals (CIs) for the association between childhood maltreatment experience (=1 and ≥ 2 *vs* 0) and specific (individual diagnoses or subtypes) endocrine diseases 20](#_Toc156565452)

[Table S7 The association of childhood maltreatment experience (≥2 *vs* <2) and the number of any or HPA-axis-related endocrine diseases 22](#_Toc156565453)

[Table S8 Hazard ratios (HRs) with 95% confidence intervals (CIs) for the association between childhood maltreatment experience (≥2 *vs* <2) with any and specific (individual diagnoses or subtypes) endocrine diseases in the cohorts for mediation analyses 23](#_Toc156565454)

[Table S9 Estimated mediated proportion of candidate mediators for the association of childhood maltreatment with any and specific endocrine diseases, using simple mediation analyses 24](#_Toc156565455)

[Table S10 Estimated mediated proportion of selected mediators, by different temporal orders, for the association between childhood maltreatment and any endocrine diseases, using sequential mediation analyses 25](#_Toc156565456)

[Table S11 Estimated mediated proportion of selected mediators, by different temporal orders, for the association between childhood maltreatment and type 2 diabetes, using sequential mediation analyses 29](#_Toc156565457)

[Table S12 Estimated mediated proportion of selected mediators, by different temporal orders, for the association between childhood maltreatment and HPA-axis-related endocrine diseases, using sequential mediation analyses 33](#_Toc156565458)

[Table S13 The competing risk of death in the relationship between childhood maltreatment and endocrine diseases 37](#_Toc156565459)

[Table S14 Sensitivity analyses for the summarized mediated proportions of four hypothesized pathways from the sequential mediation analyses 38](#_Toc156565460)

Table S1 The distribution of sociodemographic factors of UK Biobank participants

|  | **Individuals in UK Biobank** | | **Overall**  **(N=502,391)** |
| --- | --- | --- | --- |
|  | **Without childhood maltreatment information** | **With childhood maltreatment information** |  |
|  | **(N=348,798)** | **(N=153,593)** |  |
| **TDI** |  |  |  |
| Mean±SD | -1.11±3.19 | -1.71±2.83 | -1.29±3.09 |
| **Sex, No. (%)** |  |  |  |
| Female | 186,819 (53.6%) | 86,495 (56.3%) | 273,314 (54.4%) |
| Male | 161,979 (46.4%) | 67,098 (43.7%) | 229,077 (45.6%) |
| **Ethnicity, No. (%)** |  |  |  |
| White | 313,091 (89.8%) | 141,063 (91.8%) | 454,154 (90.4%) |
| Others | 33,444 (9.6%) | 12,017 (7.8%) | 45,461 (9.0%) |
| Unknown | 2,263 (0.6%) | 513 (0.3%) | 2,776 (0.6%) |
| **Place of birth, No. (%)** |  |  |  |
| England | 267,178 (76.6%) | 123,236 (80.2%) | 390,414 (77.7%) |
| Wales | 15,737 (4.5%) | 6,332 (4.1%) | 22,069 (4.4%) |
| Scotland | 28,276 (8.1%) | 11,894 (7.7%) | 40,170 (8.0%) |
| Elsewhere | 35,936 (10.3%) | 12,020 (7.8%) | 47,956 (9.5%) |
| Unknown | 1671 (0.5%) | 111 (0.1%) | 1,782 (0.4%) |
| **Number of siblings, No. (%)** |  |  |  |
| 0 | 42,761 (12.3%) | 19,392 (12.6%) | 62,153 (12.4%) |
| 1 | 104,089 (29.8%) | 53,836 (35.1%) | 157,925 (31.4%) |
| 2 | 81,732 (23.4%) | 39,634 (25.8%) | 121,366 (24.2%) |
| ≥ 3 | 117,894 (33.8%) | 40,334 (26.3%) | 158,228 (31.5%) |
| Unknown | 2,322 (0.7%) | 397 (0.3%) | 2,719 (0.5%) |
| **Maternal smoking, No. (%)** |  |  |  |
| No | 210,359 (60.3%) | 95,823 (62.4%) | 306,182 (60.9%) |
| Yes | 88,037 (25.2%) | 38,552 (25.1%) | 126,589 (25.2%) |
| Unknown | 50,402 (14.5%) | 19,218 (12.5%) | 69,620 (13.9%) |
| **Being breastfed, No. (%)** |  |  |  |
| No | 73,683 (21.1%) | 32,405 (21.1%) | 106,088 (21.1%) |
| Yes | 186,995 (53.6%) | 90,537 (58.9%) | 277,532 (55.2%) |
| Unknown | 88,120 (25.3%) | 30,651 (20.0%) | 118,771 (23.6%) |
| **Family history of diabetes, No. (%)** |  |  |  |
| No | 270,182 (77.5%) | 120,841 (78.7%) | 391,023 (77.8%) |
| Yes | 78,616 (22.5%) | 32,752 (21.3%) | 111,368 (22.2%) |
| **Education Level, No. (%)** |  |  |  |
| University | 92,318 (26.5%) | 70,218 (45.7%) | 162,536 (32.4%) |
| Non-university | 175,114 (50.2%) | 72,327 (47.1%) | 247,441 (49.3%) |
| Unknown | 81,366 (23.3%) | 11,048 (7.2%) | 92,414 (18.4%) |
| **Household income, No. (%)** |  |  |  |
| <£18,000 | 79,010 (22.7%) | 18,921 (12.3%) | 97,931 (19.5%) |
| £18,000-30,999 | 76,537 (21.9%) | 32,459 (21.1%) | 108,996 (21.7%) |
| £31,000-51,999 | 71,216 (20.4%) | 40,405 (26.3%) | 111,621 (22.2%) |
| £52,000-100,000 | 50,472 (14.5%) | 36,402 (23.7%) | 86,874 (17.3%) |
| >£100,000 | 11,897 (3.4%) | 11,169 (7.3%) | 23,066 (4.6%) |
| Unknown | 59,666 (17.1%) | 14,237 (9.3%) | 73,903 (14.7%) |
| **Employment status, No. (%)** |  |  |  |
| Employed | 310,739 (89.1%) | 143,082 (93.2%) | 453,821 (90.3%) |
| Other | 34,112 (9.8%) | 9,553 (6.2%) | 43,665 (8.7%) |
| Unknown | 3,947 (1.1%) | 958 (0.6%) | 4,905 (1.0%) |

Table S2 The definition of exposure, covariates and candidate mediators in the present study

|  | **Questions in UK Biobank** | **Recoding system and variable types** | **Note** |
| --- | --- | --- | --- |
| **Exposure** |  |  |  |
| Childhood maltreatment | 1.“When I was growing up: People in my family hit me so hard that it left me with bruises or marks” (physical abuse)  2.“When I was growing up: I felt that someone in my family hated me” (emotional abuse)  3. “When I was growing up: Someone molested me (sexually).” (sexual abuse) | No: Never true  Yes: Rarely true/ Sometimes true/ Often true/ Very often true | Summing five recorded questions to generate the cumulative number of experienced childhood maltreatment |
|  |  |  |  |
|  | 4. “When I was growing up: I felt loved” (emotional neglect) | No: Very often true/ Often true  Yes: Sometimes true/ Rarely true/ Never true |  |
|  |  |  |  |
|  | 5. “When I was growing up: There was someone to take me to the doctor if I needed it.” (physical neglect) | No: Very often true  Yes: Often true/ Sometimes true/ Rarely true/ Never true |  |
| **Covariates** |  |  |  |
| Sex | “Sex” | Female; Male |  |
| Birth year | “Year of birth” | Continuous variable |  |
| Ethnicity | “Ethnic background” | White; Other (i.e., mixed, Asian or Asian British, Black or Black British, Chinese, other ethnic group); Unknown |  |
| Country of birth | “Country of birth (UK/elsewhere)” | England; Wales; Scotland; Elsewhere; Unknown |  |
| Number of siblings | 1.“Number of full brothers”  2. “Number of adopted brothers”  3. “Number of full sisters”  4. “Number of adopted sisters”  5. “Number of older siblings” |  | Summing the total number of siblings and dividing the total number of siblings by quartile  Number = 0; Number = 1; Number = 2; Number≥ 3; Unknown |
| Maternal smoking | “Maternal smoking around birth” | No; Yes; Unknown |  |
| Being breastfed | “Breastfed as a baby” | No; Yes; Unknown |  |
| Family history of diabetes | 1. “Illnesses of father”  2. “Illnesses of mother”  3. “Illnesses of siblings” | No; Yes | At least one of the family members having a history of diabetes was defined as Yes |
| **Candidate mediators** |  |  |  |
| ***Socioeconomic status*** | | | |
| Townsend Deprivation Index | “Townsend deprivation index at recruitment” | Continuous value |  |
| Education level | “Qualifications”; “Qualifications (pilot)” | University qualifications (College or University degree); Non-university qualifications (A levels/AS levels or equivalent, O levels/GCSEs or equivalent, CSEs or equivalent, NVQ or HND or HNC or equivalent, other professional qualifications); Unknown | Combing the two items |
| Household income | “Average total household income before tax”; “Average total household income before tax (pilot)” | Less than £18,000; £18,000 to £30,999; £31,000 to £51,999; £52,000 to £100,000; > £100,000 | Combing the two items |
| Employment status | “Current employment status”; “Current employment status – corrected” | Employed (In paid employment or self-employed, retired); Unemployed/ unpaid work (Looking after home and/or family, unable to work because of sickness or disability, unemployed, doing unpaid or voluntary work, full or part-time student); Unknown | Combing and synthesizing the two items (If the two results were inconsistent, we used the corrected one) |
| ***Psychological factors*** | | | |
| Self-rated mental problem | “Seen doctor (GP) for nerves, anxiety, tension or depression”; “Seen a psychiatrist for nerves, anxiety, tension or depression” | No; Yes | At least one positive answer (yes) to either of the two questions defined as “Yes” |
| Able to confide | “Able to confide” | Less than weekly (Never or almost never, once every few months, about once a month); At least weekly (About once a week, 2-4 times a week, almost daily) |  |
| Frequency of friend/family visits | “Frequency of friend/family visits”  “Frequency of friend/family visits (pilot)” | Less than weekly (Never or almost never, once every few months, about once a month); At least weekly (About once a week, 2-4 times a week, almost daily) | Combing the two items |
| Leisure/ social activities | “Leisure/social activities” | No place (None of the above); At least one place (Sports club or gym, pub or social club, religious group, adult education class, other group activity) |  |
| ***Lifestyle factors*** | | | |
| Physical activity | “Summed MET minutes per week for all activity” | <600 MET-mins/ week: low; 600~3000 MET-mins/ week: moderate; ≥3000 MET-mins/ week: high |  |
| Smoking | “Smoking status” | Never; Ever (Previous, Current) |  |
| Alcohol | “Alcohol intake frequency” | Never/ special occasions only; Drinking (< 3 times weekly, ≥ 3 times weekly) |  |
| Diet | Total fruit and vegetable group:  1.“Cooked vegetable intake”  2. “Salad / raw vegetable intake”  3. “Fresh fruit intake”  4. “Dried fruit intake” | <3 serving/day: 0; ≥3 - <5 servings/day: 0.25;≥5 servings/day: 0.5  Amount per serving:  1 piece of fresh fruit;  5 pieces of dried fruit;  3 heaped tablespoons of cooked/ raw vegetables | Summing the total score of the four groups of key food |
|  | *p*rocessed meat group:  1. “Processed meat intake” | >4 times/week: 0; 2-4 times/week: 0.25; ≤once/week: 0.5 |  |
|  | Red meat group:  1. “Beef intake”  2. “Lamb/mutton intake”  3. “Pork intake” | >4 times/week: 0; 2-4 times/week: 0.25; ≤once/week: 0.5 |  |
|  | Whole grains group:  1. “Bread intake”  2. “Bread type”  3. “Cereal intake”  4. “Cereal type” | ≤2 serving/day: 0; >2 - <5.5 servings/day: 0.25; ≥5.5 servings/day: 0.5  Amount per serving:  1 bowl/ day of bran/oat/muesli cereal;  1 slice/ day of whole-meal/wholegrain bread) |  |
| Sleep pattern | 1.“Sleep duration” | 7-8 hours per day: 1; Less than 7 hours/ more than 8 hours per day: 0 | Summing the total score of the five sleep factors and dividing the healthy sleep score into categorical value  Healthy sleep score ≥ 4: healthy sleep pattern  Healthy sleep score ≥ 2 - <4: intermediate sleep pattern  Healthy sleep score <2: poor sleep pattern |
|  | 2. “Morning/evening person (chronotype)” | Morning/ morning than evening :1; Evening/ Evening than morning: 0 |  |
|  | 3. “Sleeplessness / insomnia” | Never/rarely: 1; Sometimes/ usually: 0 |  |
|  | 4. “Snoring” | No; Yes |  |
|  | 5. “Daytime dozing / sleeping (narcolepsy)” | Never/ rarely/ sometimes: 1; Often: 0 |  |
| Body mass index | “Body mass index (BMI)” | Continuous variable |  |
| Systolic pressure | “Systolic blood pressure, automated reading”; “Systolic blood pressure, manual reading” | Continuous variable |  |
| Diastolic pressure | “Diastolic blood pressure, automated reading”; “Diastolic blood pressure, manual reading” | Continuous variable |  |
| ***Biological biomarkers*** | | | |
| C-reactive protein | “C-reactive protein” | Continuous variable |  |
| Total protein | “Total protein” | Continuous variable |  |
| Ca | “Calcium” | Continuous variable |  |
| Vitamin D | “Vitamin D” | Continuous variable |  |
| Cholesterol | “Cholesterol” | Continuous variable |  |
| Lipoprotein A | “Lipoprotein A” | Continuous variable |  |
| High density lipoprotein | “HDL_cholesterol” | Continuous variable |  |
| Low density lipoprotein | “LDL direct” | Continuous variable |  |
| Apolipoprotein A | “Apolipoprotein A” | Continuous variable |  |
| Apolipoprotein B | “Apolipoprotein B” | Continuous variable |  |
| Triglycerides | “Triglycerides” | Continuous variable |  |
| Glucose | “Glucose” | Continuous variable |  |
| Glycosylated hemoglobin | “Glycated haemoglobin (HbA1c)” | Continuous variable |  |

Table S3 International Classification of Diseases 10th edition (ICD-10) codes used to ascertain endocrine diseases in this study

| **Endocrine diseases** | **ICD-10^*^** | **No. of cases** |
| --- | --- | --- |
| ***Any endocrine disease*** | E00-E34 | 20,885 |
| ***Individual endocrine diseases*** |  |  |
| Hypothyroidism | E00, E01, E02, E03 | 9,454 |
| Hyperthyroidism | E05 | 1,729 |
| Thyroiditis | E06 | 409 |
| Type 2 diabetes | E11 | 7,347 |
| Hypoparathyroidism | E20 | 49 |
| Hyperparathyroidism | E21 | 574 |
| Hyperfunction of pituitary gland | E22 | 207 |
| Hypofunction of pituitary gland | E23 | 286 |
| Cushing syndrome | E24 | 42 |
| Hyperaldosteronism | E26 | 44 |
| Ovarian dysfunction | E28 | 545 |
| Testicular dysfunction | E29 | 124 |
| ***Subtypes of endocrine diseases*** |  |  |
| ***By gland*** |  |  |
| Endocrine diseases of thyroid gland | E00-E07 | 11,642 |
| Endocrine diseases of pancreatic gland | E10-E16 | 8,840 |
| Endocrine diseases of parathyroid gland | E20, E21 | 613 |
| Endocrine diseases of hypothalamic-pituitary gland | E22, E23 | 468 |
| Endocrine diseases of adrenal gland | E24, E25, E26, E27 | 346 |
| Endocrine diseases of genital gland | E28, E29, E30 | 669 |
| ***By involved axis*** |  |  |
| Hypothalamic-pituitary-gonadal axis | E22, E23, E28, E29, E30 | 1,109 |
| Hypothalamic-pituitary-thyroid axis | E22, E23, E00-E07 | 11,967 |
| Hypothalamic-pituitary-adrenal axis | E22, E23, E24, E25, E26, E27 | 773 |

*****Operation, pregnancy, childbirth, puerperium, and neonate related endocrine complications, as well we endocrine complications in other systems (e.g., ophthalmic complications, neurological complications) were excluded in the present study

Table S4 Baseline characteristics of study population in the cohort for mediation analyses (n=138,498)

|  | **Different levels of childhood maltreatment experience** | | | **Overall (n=138,498)** |
| --- | --- | --- | --- | --- |
|  | **0**  **N=74,803 (54.01%)** | **1**  **N=35,383 (25.55%)** | **≥ 2**  **N=28,312 (20.44%)** |  |
| **Birth year** |  |  |  |  |
| Mean (SD) | 1950 (7.70) | 1950 (7.78) | 1950 (7.80) | 1950 (7.75) |
| **Age at the index date, year** |  |  |  |  |
| Mean (SD) | 34.6 (4.81) | 34.7 (4.92) | 34.0 (4.72) | 34.5 (4.83) |
| **Age at recruitment, year** |  |  |  |  |
| Mean (SD) | 56.5 (7.71) | 56.6 (7.79) | 55.4 (7.80) | 56.3 (7.76) |
| **Follow-up time, year** |  |  |  |  |
| Mean (SD) | 32.4 (4.03) | 32.4 (4.04) | 31.9 (4.30) | 32.3 (4.09) |
| **Sex, No. (%)** |  |  |  |  |
| Female | 40,995 (54.80) | 18,545 (52.41) | 16,632 (58.75) | 76,172 (55.00) |
| Male | 33,808 (45.20) | 16,838 (47.59) | 11,680 (41.25) | 62,326 (45.00) |
| **Place of birth, No. (%)** |  |  |  |  |
| England | 60,674 (81.11) | 28,224 (79.77) | 22,360 (78.98) | 111,258 (80.33) |
| Wales | 3,274 (4.38) | 1,373 (3.88) | 988 (3.49) | 5,635 (4.07) |
| Scotland | 5,841 (7.81) | 2,794 (7.90) | 2,086 (7.37) | 10,721 (7.74) |
| Elsewhere | 4,964 (6.64) | 2,967 (8.39) | 2,849 (10.06) | 10,780 (7.78) |
| Unknown | 50 (0.07) | 25 (0.07) | 29 (0.10) | 104 (0.08) |
| **Ethnicity, No. (%)** |  |  |  |  |
| White | 69,857 (93.39) | 32,373 (91.49) | 25,013 (88.35) | 127,243 (91.87) |
| Others | 4,731 (6.32) | 2,882 (8.15) | 3,182 (11.24) | 10,795 (7.79) |
| Unknown | 215 (0.29) | 128 (0.36) | 117 (0.41) | 460 (0.33) |
| **Number of siblings, No. (%)** |  |  |  |  |
| 0 | 10,412 (13.92) | 4,139 (11.70) | 2,816 (9.95) | 17,367 (12.54) |
| 1 | 28,469 (38.06) | 11,954 (33.78) | 8,315 (29.37) | 48,738 (35.19) |
| 2 | 19,435 (25.98) | 9,284 (26.24) | 7,219 (25.50) | 35,938 (25.95) |
| ≥ 3 | 16,319 (21.82) | 9,928 (28.06) | 9,862 (34.83) | 36,109 (26.07) |
| Unknown | 168 (0.22) | 78 (0.22) | 100 (0.35) | 346 (0.25) |
| **Maternal smoking, No. (%)** |  |  |  |  |
| No | 49,165 (65.73) | 21,905 (61.91) | 15,747 (55.62) | 86,817 (62.68) |
| Yes | 16,813 (22.48) | 8,976 (25.37) | 8,713 (30.77) | 34,502 (24.91) |
| Unknown | 8,825 (11.80) | 4,502 (12.72) | 3,852 (13.61) | 17,179 (12.40) |
| **Being breastfed, No. (%)** |  |  |  |  |
| No | 15,448 (20.65) | 7,356 (20.79) | 6,504 (22.97) | 29,308 (21.16) |
| Yes | 45,422 (60.72) | 20,708 (58.53) | 15,581 (55.03) | 81,711 (59.00) |
| Unknown | 13,933 (18.63) | 7,319 (20.69) | 6,227 (21.99) | 27,479 (19.84) |
| **Family history of diabetes, No. (%)** |  |  |  |  |
| No | 60,180 (80.45) | 28,223 (79.76) | 21,919 (77.42) | 110,322 (79.66) |
| Yes | 14,623 (19.55) | 7,160 (20.24) | 6,393 (22.58) | 28,176 (20.34) |

Abbreviation: SD, standard deviation

Table S5 Distribution of candidate mediators among individuals with different levels of childhood maltreatment experience (≥ 2 *vs* <2) in the cohorts for mediation analyses

| **Candidate mediators** | **Any endocrine diseases** | | **Type 2 diabetes** | | **HPA-axis-related endocrine disease** | |
| --- | --- | --- | --- | --- | --- | --- |
|  | **< 2**  **(n=110,186)** | **≥ 2**  **(n=28,312)** | **< 2**  **(n=118,441)** | **≥ 2**  **(n=30,949)** | **< 2**  **(n=119,854)** | **≥ 2**  **(n=31,471)** |
| **Socioeconomic status** |  |  |  |  |  |  |
| **Townsend deprivation index** |  |  |  |  |  |  |
| Mean (SD) | -1.86 (2.74) | -1.24 (3.06) | -1.85 (2.75) | -1.23 (3.07) | -1.84 (2.75) | -1.22 (3.07) |
| Missing data | 133 (0.12) | 46 (0.16) | 145 (0.12) | 48 (0.16) | 146 (0.12) | 48 (0.15) |
| **Education level** |  |  |  |  |  |  |
| University | 52,067 (47.25) | 12,147 (42.90) | 55,357 (46.74) | 13,190 (42.62) | 55,877 (46.62) | 13,334 (42.37) |
| Non-university | 50,914 (46.21) | 13,801 (48.75) | 55,108 (46.53) | 15,116 (48.84) | 55,823 (46.58) | 15,421 (49.00) |
| Missing data | 7,205 (6.54) | 2,364 (8.35) | 7,976 (6.73) | 2,643 (8.54) | 8,154 (6.80) | 2,716 (8.63) |
| **Household income** |  |  |  |  |  |  |
| <£18,000 | 12,003 (10.89) | 4,362 (15.41) | 13,276 (11.21) | 4,886 (15.79) | 13,538 (11.30) | 5,043 (16.02) |
| £18,000-30,999 | 22,723 (20.62) | 6,206 (21.92) | 24,649 (20.81) | 6,784 (21.92) | 25,049 (20.90) | 6,920 (21.99) |
| £31,000-51,999 | 29,259 (26.55) | 7,427 (26.23) | 31,309 (26.43) | 8,093 (26.15) | 31,627 (26.39) | 8,192 (26.03) |
| £52,000-100,000 | 27,352 (24.82) | 6,193 (21.87) | 28,956 (24.45) | 6,667 (21.54) | 29,188 (24.35) | 6,735 (21.40) |
| >£100,000 | 8,672 (7.87) | 1,756 (6.20) | 9,109 (7.69) | 1,860 (6.01) | 9,157 (7.64) | 1,875 (5.96) |
| Missing data | 10,177 (9.24) | 2,368 (8.36) | 11,142 (9.41) | 2,659 (8.59) | 11,295 (9.42) | 2,706 (8.60) |
| **Employment status** |  |  |  |  |  |  |
| Employed | 103,560 (93.99) | 25,734 (90.89) | 111,185 (93.87) | 28,085 (90.75) | 112,496 (93.86) | 28,525 (90.64) |
| Other | 6,013 (5.46) | 2,354 (8.31) | 6,578 (5.55) | 2,624 (8.48) | 6,672 (5.57) | 2,699 (8.58) |
| Missing data | 613 (0.56) | 224 (0.79) | 678 (0.57) | 240 (0.78) | 686 (0.57) | 247 (0.86) |
| **Psychological factors** |  |  |  |  |  |  |
| **Self-rated mental problem** |  |  |  |  |  |  |
| No | 77,319 (70.17) | 15,315 (54.09) | 82,520 (69.72) | 16,518 (53.37) | 83,479 (69.65) | 16,760 (53.26) |
| Yes | 32,372 (29.38) | 12,820 (45.28) | 35,380 (29.87) | 14,236 (46.00) | 35,823 (29.89) | 14,508 (46.10) |
| Missing data | 495 (0.45) | 177 (0.63) | 541 (0.46) | 195 (0.63) | 552 (0.46) | 203 (0.65) |
| **Able to confide** |  |  |  |  |  |  |
| < weekly | 23,264 (21.11) | 7,983 (28.20) | 24,981 (21.09) | 8,759 (28.30) | 25,338 (21.14) | 8,951 (28.44) |
| ≥ weekly | 84,315 (76.52) | 19,748 (69.75) | 90,649 (76.54) | 21,562 (69.67) | 91,652 (76.47) | 21,868 (69.49) |
| Missing data | 2,607 (2.37) | 581 (2.05) | 2,811 (2.37) | 628 (2.03) | 2,864 (2.39) | 652 (2.07) |
| **Family/ friend visit** |  |  |  |  |  |  |
| < weekly | 25,295 (22.96) | 7,689 (27.16) | 26,959 (22.76) | 8,344 (26.96) | 27,285 (22.77) | 8,490 (26.98) |
| ≥ weekly | 84,552 (76.74) | 20,520 (72.48) | 91,118 (76.93) | 22,494 (72.68) | 92,200 (76.93) | 22,867 (72.66) |
| Missing data | 339 (0.31) | 103 (0.36) | 364 (0.31) | 111 (0.36) | 369 (0.31) | 114 (0.36) |
| **Leisure/social activities** |  |  |  |  |  |  |
| No place | 29,994 (27.22) | 8,641 (30.52) | 32,399 (27.35) | 9,529 (30.79) | 32,843 (27.40) | 9,712 (30.86) |
| At least one place | 79,993 (72.60) | 19,608 (69.26) | 85,828 (72.46) | 21,351 (68.99) | 86,791 (72.41) | 21,687 (68.91) |
| Missing data | 199 (0.18) | 63 (0.22) | 214 (0.18) | 69 (0.22) | 220 (0.18) | 72 (0.23) |
| **Lifestyle factors** |  |  |  |  |  |  |
| **Physical activities** |  |  |  |  |  |  |
| High | 25,772 (23.39) | 7,132 (25.19) | 27,540 (23.325) | 7,725 (24.96) | 27,781 (23.18) | 7,824 (24.86) |
| Moderate | 51,689 (46.91) | 12,666 (44.74) | 55,332 (46.72) | 13,807 (44.61) | 55,935 (46.69) | 14,003 (44.49) |
| Low | 16,669 (15.13) | 4,366 (15.42) | 18,107 (15.29) | 4,844 (15.65) | 18,445 (15.39) | 4,979 (15.82) |
| Missing data | 16,056 (14.57) | 4,148 (14.65) | 17,462 (14.74) | 4,573 (14.78) | 17,693 (14.76) | 4,665 (14.82) |
| **Smoking** |  |  |  |  |  |  |
| Never | 66,120 (60.01) | 14,199 (50.15) | 70,758 (59.74) | 15,417 (49.81) | 71,344 (59.53) | 15,624 (49.65) |
| Ever | 43,840 (39.79) | 14,027 (49.54) | 47,433 (40.05) | 15,439 (49.89) | 48,253 (40.26) | 15,751 (50.05) |
| Missing data | 226 (0.21) | 86 (0.30) | 250 (0.21) | 93 (0.30) | 257 (0.21) | 96 (0.31) |
| **Alcohol** |  |  |  |  |  |  |
| Never/ occasions | 14,218 (12.90) | 4,931 (17.42) | 15,895 (13.42) | 5,591 (18.07) | 16,280 (13.58) | 5,787 (18.39) |
| Drinking | 95,895 (87.03) | 23,350 (82.47) | 102,469 (86.51) | 25,324 (81.82) | 103,496 (86.35) | 25,650 (81.50) |
| Missing data | 73 (0.07) | 31 (0.11) | 77 (0.07) | 34 (0.11) | 78 (0.07) | 34 (0.11) |
| **Diet** |  |  |  |  |  |  |
| Mean (SD) | 1.46 (0.37) | 1.46 (0.38) | 1.46 (0.37) | 1.46 (0.38) | 1.46 (0.37) | 1.46 (0.38) |
| Missing data | 13,599 (12.3%) | 4,474 (15.8%) | 14,598 (12.33) | 4,886 (15.79) | 14,770 (12.32) | 4,971 (15.80) |
| **Sleep pattern** |  |  |  |  |  |  |
| poor sleep | 2,873 (2.61) | 1,118 (3.95) | 3,174 (2.68) | 1,272 (4.11) | 3,251 (2.71) | 1,332 (4.23) |
| Intermediate sleep | 50,138 (45.50) | 13,973 (49.35) | 54,060 (45.64) | 15,350 (49.60) | 54,779 (45.70) | 15,616 (49.62) |
| Healthy sleep | 39,141 (35.52) | 8,467 (29.91) | 41,837 (35.32) | 9,166 (29.62) | 42,194 (35.20) | 9,261 (29.43) |
| Missing data | 18,034 (16.37) | 4,754 (16.79) | 19,370 (16.35) | 5,161 (16.76) | 19,630 (16.38) | 5,262 (16.72) |
| **Body mass index** (kg/m^2^) |  |  |  |  |  |  |
| Mean (SD) | 26.5 (4.26) | 27.0 (4.68) | 26.6 (4.37) | 27.2 (4.82) | 26.6 (4.42) | 27.3 (4.91) |
| Missing data | 264 (0.24) | 76 (0.27) | 284 (0.24) | 81 (0.26) | 290 (0.24) | 86 (0.27) |
| **Systolic pressure** (mmHg) |  |  |  |  |  |  |
| Mean (SD) | 136 (18.1) | 135 (18.0) | 136 (18.0) | 135 (18.0) | 137 (18.0) | 135 (18.0) |
| Missing data | 88 (0.08) | 29 (0.10) | 101 (0.09) | 33 (0.11) | 107 (0.09) | 34 (0.11) |
| **Diastolic pressure** (mmHg) |  |  |  |  |  |  |
| Mean (SD) | 81.8 (9.95) | 81.7 (10.1) | 81.7 (9.93) | 81.7 (10.1) | 81.7 (9.92) | 81.7 (10.1) |
| Missing data | 88 (0.08) | 29 (0.10) | 101 (0.09) | 33 (0.11) | 107 (0.09) | 34 (0.11) |
| **Biological biomarkers** |  |  |  |  |  |  |
| **C-reactive protein** (mg/L) |  |  |  |  |  |  |
| Mean (SD) | 2.17 (3.89) | 2.35 (4.00) | 2.20 (3.92) | 2.40 (4.04) | 2.21 (3.91) | 2.43 (4.08) |
| Missing data | 6,709 (6.09) | 1,780 (6.29) | 7,227 (6.10) | 1,930 (6.24) | 7,315 (6.10) | 1,962 (6.23) |
| **Total protein** (g/L) |  |  |  |  |  |  |
| Mean (SD) | 72.3 (3.99) | 72.2 (4.00) | 72.3 (4.00) | 72.2 (4.01) | 72.3 (4.00) | 72.2 (4.01) |
| Missing data | 15,497 (14.11) | 4,002 (14.14) | 16,702 (14.1%0) | 4,356 (14.07) | 16,907 (14.11) | 4,428 (14.07) |
| **Ca** (mmol/L) |  |  |  |  |  |  |
| Mean (SD) | 2.38 (0.09) | 2.38 (0.09) | 2.38 (0.09) | 2.38 (0.09) | 2.38 (0.09) | 2.38 (0.09) |
| Missing data | 15,447 (14.02) | 3,989 (14.09) | 16,645 (14.05) | 4,341 (14.03) | 16,849 (14.06) | 4,413 (14.02) |
| **Vitamin D** (nmol/L) |  |  |  |  |  |  |
| Mean (SD) | 49.9 (20.6) | 48.7 (21.0) | 49.8 (20.7) | 48.5 (21.0) | 49.8 (20.7) | 48.4 (21.0) |
| Missing data | 10,812 (9.81) | 2,890 (10.21) | 11,635 (9.82) | 3,151 (10.18) | 11,733 (9.79) | 3,188 (10.13) |
| **Cholesterol** (mmol/L) |  |  |  |  |  |  |
| Mean (SD) | 5.78 (1.09) | 5.75 (1.09) | 5.76 (1.10) | 5.73 (1.10) | 5.74 (1.11) | 5.71 (1.11) |
| Missing data | 6,520 (5.92) | 1,721 (6.08) | 7,023 (5.93) | 1,867 (6.03) | 7,111 (5.93) | 1,899 (6.03) |
| **Lipoprotein A** (nmol/L) |  |  |  |  |  |  |
| Mean (SD) | 43.8 (48.9) | 44.0 (48.9) | 43.9 (48.9) | 44.1 (48.9) | 43.9 (48.9) | 44.1 (48.9) |
| Missing data | 26,847 (24.37) | 6,858 (24.22) | 28,914 (24.41) | 7,524 (24.31) | 29,331 (24.47) | 7,664 (24.35) |
| **High density lipoprotein** (mmol/L) |  |  |  |  |  |  |
| Mean (SD) | 1.49 (0.38) | 1.48 (0.38) | 1.49 (0.38) | 1.47 (0.38) | 1.49 (0.39) | 1.47 (0.38) |
| Missing data | 15,433 (14.01) | 3,990 (14.09) | 16,626 (14.04) | 4,347 (14.05) | 16,834 (14.05) | 4,420 (14.05) |
| **Low density lipoprotein** (mmol/L) |  |  |  |  |  |  |
| Mean (SD) | 3.61 (0.83) | 3.59 (0.83) | 3.60 (0.84) | 3.58 (0.84) | 3.58 (0.84) | 3.56 (0.85) |
| Missing data | 6,725 (6.10) | 1,770 (6.25) | 7,248 (6.12) | 1,919 (6.20) | 7,342 (6.13) | 1,953 (6.21) |
| **Triglycerides** (mmol/L) |  |  |  |  |  |  |
| Mean (SD) | 1.65 (0.96) | 1.68 (1.00) | 1.65 (0.96) | 1.70 (1.01) | 1.66 (0.96) | 1.71 (1.02) |
| Missing data | 6,594 (5.98) | 1,750 (6.18) | 7,104 (6.00) | 1,899 (6.14) | 7,195 (6.00) | 1,932 (6.13) |
| **Apolipoprotein A** (g/L) |  |  |  |  |  |  |
| Mean (SD) | 1.56 (0.27) | 1.55 (0.27) | 1.56 (0.27) | 1.55 (0.27) | 1.56 (0.27) | 1.55 (0.27) |
| Missing data | 15,994 (14.52) | 4,127 (14.58) | 17,228 (14.55) | 4,495 (14.52) | 17,431 (14.54) | 4,567 (14.51) |
| **Apolipoprotein B** (g/L) |  |  |  |  |  |  |
| Mean (SD) | 1.04 (0.23) | 1.04 (0.23) | 1.04 (0.23) | 1.04 (0.23) | 1.03 (0.23) | 1.03 (0.24) |
| Missing data | 6,977 (6.33) | 1,831 (6.47) | 7,520 (6.35) | 1,994 (6.44) | 7,619 (6.36) | 2,032 (6.46) |
| **Glucose** (mmol/L) |  |  |  |  |  |  |
| Mean (SD) | 4.97 (0.74) | 4.96 (0.79) | 5.00 (0.87) | 5.01 (0.97) | 5.04 (1.00) | 5.06 (1.12) |
| Missing data | 15,497 (14.06) | 4,021 (14.20) | 16,696 (14.10) | 4,379 (14.15) | 16,902 (14.10) | 4,452 (14.15) |
| **Glycosylated hemoglobin** (mmol/mol) |  |  |  |  |  |  |
| Mean (SD) | 34.8 (4.04) | 34.8 (4.32) | 35.0 (4.65) | 35.1 (5.05) | 35.2 (5.23) | 35.4 (5.83) |
| Missing data | 7,171 (6.51) | 1,899 (6.71) | 7,702 (6.50) | 2,065 (6.67) | 7,799 (6.51) | 2,097 (6.66) |

Abbreviations: HPA: Hypothalamic-pituitary-adrenal

Table S6 Hazard ratios (HRs) with 95% confidence intervals (CIs) for the association between childhood maltreatment experience (=1 and ≥ 2 *vs* 0) and specific (individual diagnoses or subtypes) endocrine diseases

| **Specific endocrine diseases** | **Different levels of childhood maltreatment experience** | **Number of cases (incidence rate, per 1,000 person years)** | **HR (95% CI)^*^** |
| --- | --- | --- | --- |
| **Individual endocrine diseases** |  |  |  |
| Hypothyroidism | 0 | 4,752 (1.83) | Ref |
|  | 1 | 2,324 (1.88) | 1.04 (0.99-1.10) |
|  | ≥2 | 2,378 (2.41) | 1.26 (1.20-1.33) |
| Hyperthyroidism | 0 | 891 (0.34) | Ref |
|  | 1 | 409 (0.32) | 0.98 (0.87-1.10) |
|  | ≥2 | 429 (0.42) | 1.20 (1.06-1.34) |
| Thyroiditis | 0 | 206 (0.08) | Ref |
|  | 1 | 112 (0.09) | 1.16 (0.92-1.46) |
|  | ≥2 | 91 (0.09) | 1.07 (0.83-1.37) |
| Type 2 diabetes | 0 | 3,473 (1.32) | Ref |
|  | 1 | 1,950 (1.56) | 1.14 (1.08-1.21) |
|  | ≥2 | 1,924 (1.92) | 1.47 (1.39-1.55) |
| Hypoparathyroidism | 0 | 22 (0.01) | Ref |
|  | 1 | 13 (0.01) | 1.21 (0.61-2.41) |
|  | ≥2 | 14 (0.01) | 1.50 (0.76-2.97) |
| Hyperparathyroidism | 0 | 304 (0.11) | Ref |
|  | 1 | 127 (0.10) | 0.88 (0.72-1.09) |
|  | ≥2 | 143 (0.14) | 1.21 (0.99-1.49) |
| Hyperfunction of pituitary gland | 0 | 111 (0.04) | Ref |
|  | 1 | 49 (0.04) | 0.92 (0.66-1.29) |
|  | ≥2 | 47 (0.05) | 1.07 (0.76-1.52) |
| Hypofunction of pituitary gland | 0 | 133 (0.05) | Ref |
|  | 1 | 75 (0.06) | 1.16 (0.87-1.54) |
|  | ≥2 | 78 (0.08) | 1.48 (1.11-1.97) |
| Cushing syndrome | 0 | 19 (0.01) | Ref |
|  | 1 | 12 (0.01) | 1.36 (0.66-2.82) |
|  | ≥2 | 11 (0.01) | 1.51 (0.71-3.21) |
| Hyperaldosteronism | 0 | 29 (0.01) | Ref |
|  | 1 | 7 (0.01) | 0.53 (0.23-1.21) |
|  | ≥2 | 8 (0.01) | 0.79 (0.36-1.75) |
| Ovarian dysfunction | 0 | 258 (0.10) | Ref |
|  | 1 | 140 (0.11) | 1.20 (0.98-1.48) |
|  | ≥2 | 147 (0.14) | 1.23 (1.00-1.51) |
| Testicular dysfunction | 0 | 64 (0.02) | Ref |
|  | 1 | 39 (0.03) | 1.24 (0.83-1.85) |
|  | ≥2 | 21 (0.02) | 0.93 (0.56-1.53) |
| **Subtypes of endocrine diseases** |  |  |  |
| ***By involved gland*** |  |  |  |
| Endocrine disorders of thyroid gland | 0 | 5,912 (2.29) | Ref |
|  | 1 | 2,860 (2.33) | 1.03 (0.99-1.08) |
|  | ≥2 | 2,870 (2.93) | 1.22 (1.17-1.28) |
| Endocrine disorders of pancreatic gland | 0 | 4,233 (1.62) | Ref |
|  | 1 | 2,349 (1.89) | 1.13 (1.07-1.19) |
|  | ≥2 | 2,258 (2.27) | 1.41 (1.34-1.49) |
| Endocrine disorders of parathyroid gland | 0 | 320 (0.12) | Ref |
|  | 1 | 140 (0.11) | 0.93 (0.76-1.13) |
|  | ≥2 | 153 (0.15) | 1.23 (1.01-1.50) |
| Endocrine diseases of hypothalamic-pituitary gland | 0 | 229 (0.09) | Ref |
|  | 1 | 118 (0.09) | 1.07 (0.86-1.34) |
|  | ≥2 | 121 (0.12) | 1.34 (1.07-1.67) |
| Endocrine diseases of adrenal gland | 0 | 170 (0.06) | Ref |
|  | 1 | 75 (0.06) | 0.92 (0.70-1.21) |
|  | ≥2 | 101 (0.10) | 1.50 (1.17-1.93) |
| Endocrine diseases of genital gland | 0 | 322 (0.12) | Ref |
|  | 1 | 179 (0.14) | 1.21 (1.01-1.45) |
|  | ≥2 | 168 (0.17) | 1.19 (0.98-1.44) |
| ***By involved axis*** |  |  |  |
| Hypothalamic-pituitary-gonadal axis | 0 | 540 (0.20) | Ref |
|  | 1 | 285 (0.23) | 1.13 (0.98-1.30) |
|  | ≥2 | 284 (0.28) | 1.25 (1.08-1.45) |
| Hypothalamic-pituitary-thyroid axis | 0 | 6,075 (2.36) | Ref |
|  | 1 | 2,944 (2.40) | 1.03 (0.99-1.08) |
|  | ≥2 | 2,948 (3.02) | 1.22 (1.17-1.27) |
| Hypothalamic-pituitary-adrenal axis | 0 | 386 (0.15) | Ref |
|  | 1 | 179 (0.14) | 0.96 (0.81-1.15) |
|  | ≥2 | 208 (0.20) | 1.36 (1.15-1.62) |

^*^HRs and 95% CIs were derived from Cox models, adjusting for sex, birth year, ethnicity, country of birth, number of siblings, maternal smoking, being breastfed and family history of diabetes

Table S7 The association of childhood maltreatment experience (≥2 *vs* <2) and the number of any or HPA-axis-related endocrine diseases

|  | **Number of cases (%)** | | **Odds ratios (95% confidence interval) *** |
| --- | --- | --- | --- |
|  | **Exposed individuals (≥2)** | **Unexposed individuals (<2)** |  |
| ***The number of any endocrine diseases*** |  |  |  |
| As continuous variable, mean (SD) | 0.23(0.60) | 0.18(0.52) | 1.05(1.04-1.06) |
| As categorized variable, |  |  |  |
| 0 | 26,380(83.6%) | 104,394(86.9%) | Ref |
| 1 | 3,495(11.1) | 10,891(9.10) | 1.24(1.19-1.30) |
| 2 | 1,322(4.2%) | 3,909(3.3%) | 1.35(1.27-1.44) |
| ≥3 | 362(1.1%) | 906(0.8%) | 1.52(1.52-1.53) |
| ***The number of HPA-axis endocrine diseases*** |  |  |  |
| As continuous variable, mean (SD) | 0.01(0.10) | 0.01(0.08) | 1.00(1.00-1.00) |
| As categorized variable |  |  |  |
| 0 | 31,351(99.3%) | 119,535(99.5%) | Ref |
| 1 | 186(0.6%) | 506(0.4%) | 1.38(1.16-1.63) |
| 2 | 20(0.1%) | 56(0.0%) | 1.36(1.36-1.36) |
| ≥3 | 2(0.0%) | 3(0.0%) | 1.91(1.91-1.91) |

*Odds ratios (95% confidence interval) were derived from multinomial logistic regression models, adjusted for sex, birth year, ethnicity, country of birth, number of siblings, maternal smoking, being breastfed, and family history of diabetes

Table S8 Hazard ratios (HRs) with 95% confidence intervals (CIs) for the association between childhood maltreatment experience (≥2 *vs* <2) with any and specific (individual diagnoses or subtypes) endocrine diseases in the cohorts for mediation analyses

| **Any or specific endocrine diseases** | **Number of cases**  **(Incidence rate, per 1,000 person years)**  **(≥ 2 *vs* < 2)** | **HR (95% CI)^*^** |
| --- | --- | --- |
| **Any endocrine diseases** |  |  |
|  | 1932(2.14)/ 5792(1.62) | 1.30 (1.24-1.37) |
| **Individual endocrine diseases** |  |  |
| Hypothyroidism | 737 (0.77)/ 2,130 (0.57) | 1.30 (1.19-1.41) |
| Hyperthyroidism | 153 (0.15)/ 428 (0.11) | 1.31 (1.09-1.58) |
| Thyroiditis | 39 (0.04)/ 99 (0.03) | 1.37 (0.94-1.99) |
| Type 2 diabetes | 1,314 (1.32)/ 3,764 (0.98) | 1.38 (1.29-1.47) |
| Hypoparathyroidism | 8 (0.01)/ 22(0.01) | 1.31 (0.58-2.99) |
| Hyperparathyroidism | 103 (0.10)/ 310 (0.08) | 1.27 (1.01-1.59) |
| Hyperfunction of pituitary gland | 27 (0.03)/ 88 (0.02) | 1.24 (0.80-1.93) |
| Hypofunction of pituitary gland | 40 (0.04)/ 101 (0.03) | 1.46 (1.00-2.11) |
| Cushing syndrome | 5 (0.00)/ 12 (0.00) | 1.61 (0.56-4.63) |
| Hyperaldosteronism | 6 (0.01)/ 21 (0.01) | 1.33 (0.53-3.33) |
| Ovarian dysfunction | 22 (0.02)/ 55(0.01) | 1.17 (0.71-1.93) |
| Testicular dysfunction | 10 (0.01)/ 58 (0.01) | 0.70 (0.36-1.38) |
| **Subtypes of endocrine diseases** |  |  |
| ***By involved gland*** |  |  |
| Endocrine diseases of thyroid gland | 895 (0.94)/ 2,651 (0.71) | 1.26 (1.17-1.36) |
| Endocrine diseases of pancreatic gland | 1,086 (1.11)/ 3,124 (0.82) | 1.37 (1.28-1.47) |
| Endocrine diseases of parathyroid gland | 108 (0.11)/ 328 (0.08) | 1.26 (1.01-1.57) |
| Endocrine diseases of hypothalamic-pituitary gland | 65 (0.06)/ 180 (0.05) | 1.40 (1.05-1.86) |
| Endocrine diseases of adrenal gland | 64 (0.06)/ 158 (0.04) | 1.55 (1.15-2.08) |
| Endocrine diseases of genital gland | 32 (0.03)/ 113 (0.03) | 0.97 (0.65-1.44) |
| ***By involved axis*** |  |  |
| Hypothalamic-pituitary-gonadal axis | 94(0.09)/ 279 (0.07) | 1.25 (0.98-1.58) |
| Hypothalamic-pituitary-thyroid axis | 933 (0.98)/ 2,776 (0.75) | 1.26 (1.17-1.36) |
| Hypothalamic-pituitary-adrenal axis | 120 (0.12)/ 319 (0.08) | 1.44 (1.17-1.79) |

^*^ HRs and 95% CIs were derived from Cox models, adjusting for sex, birth year, ethnicity, country of birth, number of siblings, maternal smoking, being breastfed and family history of diabetes

Table S9 Estimated mediated proportion of candidate mediators for the association of childhood maltreatment with any and specific endocrine diseases, using simple mediation analyses

| **Candidate mediators** | **Mediated proportion (%)^*^** | | | | |
| --- | --- | --- | --- | --- | --- |
|  | **Any endocrine diseases** |  | **Type 2 diabetes** |  | **HPA-related endocrine diseases** |
| **Socioeconomic status** |  |  |  |  |  |
| Townsend Deprivation Index | 9.14 (6.02, 13.19) |  | 13.50 (10.34, 18.06) |  | 8.21 (0.06, 23.29) |
| Education level | 3.01 (1.68, 4.81) |  | 3.33 (2.03, 5.11) |  | 0.29 (-3.15, 4.63) |
| Household income | 5.26 (2.63, 12.02) |  | 3.94 (2.35, 5.81) |  | -0.37 (-8.10, 4.95) |
| Employment status | 5.72 (3.53, 7.44) |  | 5.69 (3.39, 8.13) |  | 11.64 (6.34, 33.16) |
| **Psychological factors** |  |  |  |  |  |
| Self-rated mental problem | 20.06 (14.11, 27.17) |  | 17.59 (11.92, 24.48) |  | 27.99 (10.43, 69.88) |
| Able to confide | 3.05 (0.09, 6.36) |  | 4.59 (1.90, 8.68) |  | 9.05 (-0.31, 27.75) |
| Frequency of friend/family visits | -0.79 (-2.09, 0.70) |  | -0.19 (-2.05, 1.80) |  | 0.41 (-5.01, 6.52) |
| Leisure/ social activities | 2.72 (1.76, 4.55) |  | 2.98 (1.40, 4.72) |  | 0.40 (-4.33, 4.80) |
| **Lifestyle factors** |  |  |  |  |  |
| Physical activities | 3.02 (1.64, 4.04) |  | 3.35 (2.14, 4.78) |  | 2.49 (-0.82, 8.52) |
| Smoking | 9.93 (5.30, 14.91) |  | 15.15 (10.79, 20.65) |  | 0.87 (-10.04, 13.88) |
| Alcohol | 9.71 (5.15, 14.96) |  | 10.59 (7.89, 13.46) |  | 11.85 (4.77, 29.56) |
| Diet | 0.38 (-0.53, 1.27) |  | 0.43 (-0.30, 1.27) |  | -0.23 (-2.21, 0.46) |
| Sleep pattern | 6.45 (3.68, 9.50) |  | 8.44 (6.12, 11.74) |  | 9.44 (-0.64, 52.79) |
| Body mass index | 21.45 (18.51, 26.06) |  | 30.23 (23.87, 38.55) |  | 5.96 (-0.04, 21.56) |
| Systolic pressure | -1.36 (-2.14, -0.77) |  | -2.02 (-3.27, -0.98) |  | 1.88 (-0.09, 3.68) |
| Diastolic pressure | 0.83 (0.19, 1.46) |  | 1.12 (0.44, 2.19) |  | -1.22 (-3.25, 0.02) |
| **Biological biomarkers** |  |  |  |  |  |
| C-reactive protein | 2.66 (1.82, 3.63) |  | 2.97 (2.17, 4.03) |  | 2.08 (-1.23, 6.80) |
| Total protein | -0.70 (-1.52, 0.08) |  | -0.70 (-1.66, -0.03) |  | 0.32 (-3.95, 3.43) |
| Ca | -0.81 (-1.51, -0.26) |  | -0.71 (-1.47, -0.20) |  | -0.90 (-4.23, 0.32) |
| Vitamin D | 2.80 (1.36, 4.46) |  | 5.34 (3.63, 7.44) |  | 0.74 (-2.44, 4.10) |
| Cholesterol | 1.21 (0.50, 2.41) |  | 2.91 (1.07, 4.91) |  | 1.63 (0.34, 5.39) |
| Lipoprotein A | -0.04 (-0.39, 0.26) |  | -0.01 (-0.21, 0.13) |  | -0.10 (-1.44, 0.89) |
| High density lipoprotein | 11.87 (8.20, 15.77) |  | 20.09 (16.34, 24.38) |  | 4.53 (0.04, 13.77) |
| Low density lipoprotein | 0.46 (-0.01, 1.03) |  | 1.29 (-0.27, 2.76) |  | 1.18 (0.14, 3.77) |
| Apolipoprotein A | 7.73 (5.29, 10.88) |  | 12.48 (9.68, 15.46) |  | 2.71 (-2.29, 9.24) |
| Apolipoprotein B | -0.01 (-0.26, 0.27) |  | -0.46 (-1.38, 0.20) |  | -0.29 (-1.56, 0.84) |
| Triglycerides | 7.13 (5.37, 9.78) |  | 9.56 (7.37, 11.76) |  | 5.00 (1.35, 12.13) |
| Glucose | 0.42 (-0.52, 1.50) |  | 1.13 (0.55, 2.68) |  | 0.07 (-2.74, 1.92) |
| Glycosylated hemoglobin | 3.97 (2.34, 5.97) |  | - 1. (3.70, 8.20) |  | 1.84 (-0.18, 6.83) |

*Mediation proportions was derived from Cox models, adjusting for sex, birth year, ethnicity, country of birth, number of siblings, maternal smoking, being breastfed and family history of diabetes

Table S10 Estimated mediated proportion of selected mediators, by different temporal orders, for the association between childhood maltreatment and any endocrine diseases, using sequential mediation analyses

|  | **Direct effect** | **Indirect effect** | | | |
| --- | --- | --- | --- | --- | --- |
|  |  | **M1** | **M2** | **M3** | **M4** |
| **childhood maltreatment→ socioeconomic status→ psychological factors → lifestyle factors → biological biomarkers** | | | | | |
| Mediated proportion | 41.91% (28.85%, 52.40%) | 6.24% (4.74%, 8.19%) | 39.04% (29.83%, 49.53%) | 11.90% (9.48%, 15.18%) | 0.91% (-0.09%, 1.97%) |
| *P* | <0.001 | <0.001 | <0.001 | <0.001 | 0.079 |
| **childhood maltreatment→ socioeconomic status→ psychological factors → biological biomarkers → lifestyle factors** | | | | | |
| Mediated proportion | 41.97% (28.89%, 52.47%) | 6.25% (4.74%, 8.20%) | 38.95% (29.72%, 49.45%) | 4.72% (3.10%, 6.69%) | 8.12% (6.30%, 10.54%) |
| *P* | <0.001 | <0.001 | <0.001 | <0.001 | 0.087 |
| **childhood maltreatment→ socioeconomic status → lifestyle factors → biological biomarkers→ psychological factors** | | | | | |
| Mediated proportion | 44.95% (31.28%, 55.90%) | 6.48% (4.86%, 8.63%) | 14.83% (11.89%, 18.98%) | 1.54% (0.44%, 2.78%) | 32.19% (22.64%, 42.67%) |
| *P* | <0.001 | <0.001 | <0.001 | 0.008 | <0.001 |
| **childhood maltreatment→ socioeconomic status → lifestyle factors → psychological factors→ biological biomarkers** | | | | | |
| Mediated proportion | 44.12% (30.63%, 54.97%) | 6.41% (4.83%, 8.50%) | 14.60% (11.73%, 18.62%) | 33.91% (24.46%, 44.37%) | 0.96% (-0.09%, 2.08%) |
| *P* | <0.001 | <0.001 | <0.001 | <0.001 | 0.079 |
| **childhood maltreatment→ socioeconomic status→ biological biomarkers → lifestyle factors→ psychological factors** | | | | | |
| Mediated proportion | 44.97% (31.32%, 55.93%) | 6.48% (4.87%, 8.64%) | 6.47% (4.58%, 8.89%) | 9.87% (7.68%, 12.83%) | 32.21% (22.66%, 42.71%) |
| *P* | <0.001 | <0.001 | <0.001 | <0.001 | <0.001 |
| **childhood maltreatment→ socioeconomic status → biological biomarkers → psychological factors → lifestyle factors** | | | | | |
| Mediated proportion | 43.66% (30.26%, 54.43%) | 6.38% (4.82%, 8.45%) | 6.31% (4.48%, 8.63%) | 35.21% (25.76%, 45.73%) | 8.44% (6.52%, 11.04%) |
| *P* | <0.001 | <0.001 | <0.001 | <0.001 | <0.001 |
| **childhood maltreatment→ psychological factors→ socioeconomic status → lifestyle factors → biological biomarkers** | | | | | |
| Mediated proportion | 41.23% (28.31%, 51.63%) | 41.68% (32.33%, 52.42%) | 4.48% (3.25%, 6.07%) | 11.71% (9.34%, 14.89%) | 0.89% (-0.09%, 1.94%) |
| *P* | <0.001 | <0.001 | <0.001 | <0.001 | 0.087 |
| **childhood maltreatment→ psychological factors → socioeconomic status→ biological biomarkers → lifestyle factors** | | | | | |
| Mediated proportion | 41.29% (28.34%, 51.72%) | 41.59% (32.22%, 52.34%) | 4.49% (3.26%, 6.09%) | 4.64% (3.05%, 6.56%) | 7.99% (6.21%, 10.34%) |
| *P* | <0.001 | <0.001 | <0.001 | <0.001 | <0.001 |
| **childhood maltreatment→ psychological factors → lifestyle factors→ socioeconomic status → biological biomarkers** | | | | | |
| Mediated proportion | 41.23% (28.31%, 51.65%) | 41.66% (32.30%, 52.39%) | 12.62% (10.14%, 15.99%) | 3.60% (2.48%, 5.00%) | 0.89% (-0.09%, 1.94%) |
| *P* | <0.001 | <0.001 | <0.001 | <0.001 | 0.079 |
| **childhood maltreatment→ psychological factors→ lifestyle factors→ biological biomarkers→ socioeconomic status** | | | | | |
| Mediated proportion | 41.29% (28.34%, 51.72%) | 41.69% (32.32%, 52.43%) | 12.64% (10.15%, 16.02%) | 0.85% (-0.13%, 1.89%) | 3.53% (2.44%, 4.92%) |
| *P* | <0.001 | <0.001 | <0.001 | 0.094 | <0.001 |
| **childhood maltreatment→ psychological factors→ biological biomarkers → lifestyle factors →socioeconomic status** | | | | | |
| Mediated proportion | 41.38% (28.41%, 51.81%) | 41.60% (32.20%, 52.39%) | 4.80% (3.20%, 6.75%) | 8.68% (6.80%, 11.18%) | 3.54% (2.44%, 4.92%) |
| *P* | <0.001 | <0.001 | <0.001 | <0.001 | <0.001 |
| **childhood maltreatment→ psychological factors→ biological biomarkers→ socioeconomic status→ lifestyle factors** | | | | | |
| Mediated proportion | 41.37% (28.40%, 51.79%) | 41.62% (32.23%, 52.39%) | 4.80% (3.20%, 6.75%) | 4.21% (3.01%, 5.74%) | 8.00% (6.22%, 10.36%) |
| *P* | <0.001 | <0.001 | <0.001 | <0.001 | <0.001 |
| **childhood maltreatment→ lifestyle factors→ psychological factors→ socioeconomic status→ biological biomarkers** | | | | | |
| Mediated proportion | 43.52% (30.12%, 54.25%) | 15.61% (12.63%, 19.79%) | 36.14% (26.60%, 46.79%) | 3.80% (2.61%, 5.31%) | 0.94% (-0.09%, 2.05%) |
| *P* | <0.001 | <0.001 | <0.001 | <0.001 | 0.079 |
| **childhood maltreatment→ lifestyle factors→ psychological factors→ biological biomarkers→ socioeconomic status** | | | | | |
| Mediated proportion | 43.58% (30.17%, 54.32%) | 15.63% (12.64%, 19.83%) | 36.16% (26.61%, 46.85%) | 0.90% (-0.14%, 2.00%) | 3.73% (2.57%, 5.23%) |
| *P* | <0.001 | <0.001 | <0.001 | 0.094 | <0.001 |
| **childhood maltreatment→ lifestyle factors → socioeconomic status→ psychological factors→ biological biomarkers** | | | | | |
| Mediated proportion | 44.11% (30.62%, 54.96%) | 15.80% (12.76%, 20.10%) | 5.22% (3.81%, 7.05%) | 33.91% (24.45%, 44.35%) | 0.96% (-0.09%, 2.08%) |
| *P* | <0.001 | <0.001 | <0.001 | <0.001 | 0.079 |
| **childhood maltreatment→ lifestyle factors→ socioeconomic status→ biological biomarkers→ psychological factors** | | | | | |
| Mediated proportion | 44.94% (31.28%, 55.88%) | 16.06% (12.93%, 20.49%) | 5.27% (3.83%, 7.15%) | 1.54% (0.44%, 2.78%) | 32.19% (22.65%, 42.67%) |
| *P* | <0.001 | <0.001 | <0.001 | 0.008 | <0.001 |
| **childhood maltreatment→ lifestyle factors→ biological biomarkers→ socioeconomic status→ psychological factors** | | | | | |
| Mediated proportion | 45.01% (31.35%, 55.97%) | 16.08% (12.95%, 20.53%) | 1.49% (0.40%, 2.71%) | 5.17% (3.75%, 7.03%) | 32.24% (22.68%, 42.76%) |
| *P* | <0.001 | <0.001 | 0.010 | 0.012 | <0.001 |
| **childhood maltreatment→ lifestyle factors→ biological biomarkers→ psychological factors→ socioeconomic status** | | | | | |
| Mediated proportion | 44.39% (30.84%, 55.26%) | 15.88% (12.81%, 20.22%) | 1.47% (0.39%, 2.67%) | 34.46% (24.78%, 45.18%) | 3.80% (2.61%, 5.34%) |
| *P* | <0.001 | <0.001 | 0.010 | <0.001 | <0.001 |
| **childhood maltreatment→ biological biomarkers→ lifestyle factors→ socioeconomic status→ psychological factors** | | | | | |
| Mediated proportion | 45.06% (31.38%, 56.02%) | 6.71% (4.79%, 9.17%) | 10.78% (8.50%, 13.97%) | 5.18% (3.76%, 7.04%) | 32.27% (22.70%, 42.80%) |
| *P* | <0.001 | <0.001 | <0.001 | <0.001 | <0.001 |
| **childhood maltreatment→ biological biomarkers→ lifestyle factors→ psychological factors→ socioeconomic status** | | | | | |
| Mediated proportion | 44.43% (30.88%, 55.31%) | 6.63% (4.74%, 9.03%) | 10.65% (8.41%, 13.76%) | 34.50% (24.80%, 45.24%) | 3.80% (2.61%, 5.35%) |
| *P* | <0.001 | <0.001 | <0.001 | <0.001 | <0.001 |
| **childhood maltreatment→ biological biomarkers→ psychological factors→ lifestyle factors→ socioeconomic status** | | | | | |
| Mediated proportion | 43.04% (29.75%, 53.69%) | 6.45% (4.62%, 8.74%) | 37.80% (28.27%, 48.60%) | 9.03% (7.05%, 11.70%) | 3.68% (2.54%, 5.16%) |
| *P* | <0.001 | <0.001 | <0.001 | <0.001 | <0.001 |
| **childhood maltreatment→ biological biomarkers→ psychological factors→ socioeconomic status→ lifestyle factors** | | | | | |
| Mediated proportion | 43.04% (29.74%, 53.69%) | 6.45% (4.62%, 8.74%) | 37.82% (28.29%, 48.61%) | 4.38% (3.13%, 6.01%) | 8.32% (6.44%, 10.86%) |
| *P* | <0.001 | <0.001 | <0.001 | <0.001 | <0.001 |
| **childhood maltreatment→ biological biomarkers→ socioeconomic status→ psychological factors→ lifestyle factors** | | | | | |
| Mediated proportion | 43.74% (30.32%, 54.53%) | 6.54% (4.68%, 8.90%) | 5.98% (4.48%, 7.97%) | 35.28% (25.81%, 45.840%) | 8.46% (6.54%, 11.07%) |
| *P* | <0.001 | <0.001 | <0.001 | <0.001 | <0.001 |
| **childhood maltreatment→ biological biomarkers→ socioeconomic status→ lifestyle factors→ psychological factors** | | | | | |
| Mediated proportion | 45.06% (31.38%, 56.03%) | 6.71% (4.79%, 9.17%) | 6.07% (4.52%, 8.14%) | 9.89% (7.73%, 12.86%) | 32.27% (22.70%, 42.81%) |
| *P* | <0.001 | <0.001 | <0.001 | <0.001 | <0.001 |

Abbreviations: M1: M1-mediated effect; M2: M2-mediated effect; M3: M3-mediated effect; M4: M4-mediated effect

| Table S11 Estimated mediated proportion of selected mediators, by different temporal orders, for the association between childhood maltreatment and type 2 diabetes, using sequential mediation analyses | | | | | |
| --- | --- | --- | --- | --- | --- |
|  | **Direct effect** | **Indirect effect** | | | |
|  |  | **M1** | **M2** | **M3** | **M4** |
| **childhood maltreatment→ socioeconomic status→ psychological factors → lifestyle factors →biological biomarkers** | | | | | |
| Mediated proportion | 37.73% (25.52%, 47.70%) | 6.43% (5.04%, 8.22%) | 34.65% (26.13%, 43.86%) | 18.40% (15.07%, 22.81%) | 2.79% (1.09%, 4.61%) |
| *P* | <0.001 | <0.001 | <0.001 | <0.001 | 0.002 |
| **childhood maltreatment→ socioeconomic status→ psychological factors → biological biomarkers → lifestyle factors** | | | | | |
| Mediated proportion | 37.73% (25.51%, 47.69%) | 6.43% (5.04%, 8.22%) | 34.65% (26.13%, 43.87%) | 10.12% (7.53%, 13.28%) | 11.07% (8.90%, 13.91%) |
| *P* | <0.001 | <0.001 | <0.001 | <0.001 | <0.001 |
| **childhood maltreatment→ socioeconomic status→ lifestyle factors → biological biomarkers→ psychological factors** | | | | | |
| Mediated proportion | 42.34% (29.12%, 53.06%) | 6.89% (5.32%, 9.00%) | 23.62% (19.31%, 29.67%) | 4.11% (2.18%, 6.31%) | 23.04% (13.59%, 32.52%) |
| *P* | <0.001 | <0.001 | <0.001 | <0.001 | <0.001 |
| **childhood maltreatment→ socioeconomic status→ lifestyle factors →psychological factors→ biological biomarkers** | | | | | |
| Mediated proportion | 40.98% (28.06%, 51.51%) | 6.76% (5.24%, 8.77%) | 22.92% (18.82%, 28.61%) | 26.31% (17.20%, 35.66%) | 3.03% (1.18%, 5.05%) |
| *P* | <0.001 | <0.001 | <0.001 | <0.001 | 0.002 |
| **childhood maltreatment→ socioeconomic status→ biological biomarkers → lifestyle factors→ psychological factors** | | | | | |
| Mediated proportion | 42.34% (29.14%, 53.08%) | 6.89% (5.32%, 9.00%) | 13.69% (1051%, 17.84%) | 14.04% (11.30%, 17.84%) | 23.04% (13.60%, 32.52%) |
| *P* | <0.001 | <0.001 | <0.001 | <0.001 | <0.001 |
| **childhood maltreatment→ socioeconomic status→ biological biomarkers→ psychological factors → lifestyle factors** | | | | | |
| Mediated proportion | 40.42% (27.62%, 50.85%) | 6.70% (5.21%, 8.67%) | 13.11% (10.10%, 16.95%) | 27.92% (18.88%, 37.29%) | 11.85% (9.46%, 15.07%) |
| *P* | <0.001 | <0.001 | <0.001 | <0.001 | <0.001 |
| **childhood maltreatment→ psychological factors→ socioeconomic status → lifestyle factors →biological biomarkers** | | | | | |
| Mediated proportion | 37.02% (24.97%, 46.89%) | 37.37% (28.77%, 46.75%) | 4.83% (3.67%, 6.30%) | 18.05% (14.81%, 22.31%) | 2.73% (1.07%, 4.52%) |
| *P* | <0.001 | <0.001 | <0.001 | <0.001 | 0.002 |
| **childhood maltreatment→ psychological factors→ socioeconomic status→ biological biomarkers→ lifestyle factors** | | | | | |
| Mediated proportion | 37.02% (24.96%, 46.90%) | 37.37% (28.78%, 46.75%) | 4.83% (3.67%, 6.31%) | 9.93% (7.40%, 12.99%) | 10.86% (8.74%, 13.60%) |
| *P* | <0.001 | <0.001 | <0.001 | <0.001 | <0.001 |
| **childhood maltreatment→ psychological factors→ lifestyle factors→ socioeconomic status→ biological biomarkers** | | | | | |
| Mediated proportion | 37.02% (24.96%, 46.91%) | 37.37% (28.78%, 46.76%) | 19.27% (15.89%, 23.74%) | 3.60% (2.60%, 4.85%) | 2.73% (1.07%, 4.52%) |
| *P* | <0.001 | <0.001 | <0.001 | <0.001 | 0.002 |
| **childhood maltreatment→ psychological factors→ lifestyle factors → biological biomarkers→ socioeconomic status** | | | | | |
| Mediated proportion | 37.02% (24.96%, 46.91%) | 37.37% (28.78%, 46.76%) | 19.27% (15.89%, 23.75%) | 2.63% (0.97%, 4.41%) | 3.71% (2.70%, 4.97%) |
| *P* | <0.001 | <0.001 | <0.001 | 0.002 | 0.002 |
| **childhood maltreatment→ psychological factors→ biological biomarkers→ lifestyle factors→ socioeconomic status** | | | | | |
| Mediated proportion | 37.02% (24.96%, 46.89%) | 37.37% (28.76%, 46.75%) | 10.19% (7.64%, 13.29%) | 11.71% (9.49%, 14.62%) | 3.71% (2.70%, 4.97%) |
| *P* | <0.001 | <0.001 | <0.001 | 0.002 | <0.001 |
| **childhood maltreatment→ psychological factors→ biological biomarkers→ socioeconomic status→ lifestyle factors** | | | | | |
| Mediated proportion | 37.02% (24.96%, 46.88%) | 37.02% (28.77%, 46.75%) | 10.19% (7.64%, 13.29%) | 4.57% (3.44%,6.00%) | 10.86% (8.74%, 13.61%) |
| *P* | <0.001 | <0.001 | <0.001 | <0.001 | <0.001 |
| **childhood maltreatment→ lifestyle factors→ psychological factors→ socioeconomic status→ biological biomarkers** | | | | | |
| Mediated proportion | 40.35% (27.54%, 50.75%) | 24.22% (19.99%,30.07%) | 28.53% (19.43%, 38.00%) | 3.93% (2.82%, 5.35%) | 2.98% (1.16%, 4.96%) |
| *P* | <0.001 | <0.001 | <0.001 | <0.001 | 0.002 |
| **childhood maltreatment→ lifestyle factors→ psychological factors→ biological biomarkers→ socioeconomic status** | | | | | |
| Mediated proportion | 40.35% (27.54%, 50.75%) | 24.22% (19.98%, 30.06%) | 28.53% (19.43%, 38.00%) | 2.87% (1.06%, 4.84%) | 4.04% (2.93%, 5.48%) |
| *P* | <0.001 | <0.001 | <0.001 | 0.003 | <0.001 |
| **childhood maltreatment→ lifestyle factors→ socioeconomic status→ psychological factors→ biological biomarkers** | | | | | |
| Mediated proportion | 40.98% (28.05%, 51.50%) | 24.57% (20.23%, 30.61%) | 5.11% (3.82%, 6.78%) | 26.31% (18.00%. 35.66%) | 3.03% (1.18%, 5.05%) |
| *P* | <0.001 | <0.001 | <0.001 | <0.001 | 0.002 |
| **childhood maltreatment→ lifestyle factors→ socioeconomic status→ biological biomarkers→ psychological factors** | | | | | |
| Mediated proportion | 42.34% (29.12%, 53.07%) | 25.32% (20.76%, 31.75%) | 5.20% (3.87%, 6.95%) | 4.11% (2.18%, 6.31%) | 23.04% (13.59%, 32.52%) |
| *P* | <0.001 | <0.001 | <0.001 | <0.001 | <0.001 |
| **childhood maltreatment→ lifestyle factors→ biological biomarkers→ socioeconomic status→ psychological factors** | | | | | |
| Mediated proportion | 42.34% (29.12%, 53.07%) | 25.32% (20.77%, 31.75%) | 3.99% (2.08%, 6.17%) | 5.32% (3.97%, 7.08%) | 23.04% (13.60%, 32.51%) |
| *P* | <0.001 | <0.001 | <0.001 | <0.001 | <0.001 |
| **childhood maltreatment→ lifestyle factors→ biological biomarkers→ psychological factors→ socioeconomic status** | | | | | |
| Mediated proportion | 41.64% (28.57%, 52.26%) | 24.93% (20.50%, 31.16%) | 3.93% (2.05%, 6.06%) | 25.33% (15.87%, 34.93%) | 4.17% (3.01%, 5.68%) |
| *P* | <0.001 | <0.001 | <0.001 | <0.001 | <0.001 |
| **childhood maltreatment→ biological biomarkers→ lifestyle factors→ socioeconomic status→ psychological factors** | | | | | |
| Mediated proportion | 42.34% (29.12%, 53.08%) | 14.09% (10.87%, 18.29%) | 15.22% (12.31%, 19.28%) | 5.32% (3.97%, 7.08%) | 23.04% (13.60%, 32.51%) |
| *P* | <0.001 | <0.001 | <0.001 | <0.001 | <0.001 |
| **childhood maltreatment→ biological biomarkers→ lifestyle factors→ psychological factors→ socioeconomic status** | | | | | |
| Mediated proportion | 41.64% (28.58%, 52.27%) | 13.87% (10.72%, 17.96%) | 14.99% (12.15%, 18.93%) | 25.33% (15.88%, 34.91%) | 4.17% (3.01%, 5.69%) |
| *P* | <0.001 | <0.001 | <0.001 | <0.001 | 0.011 |
| **childhood maltreatment→ biological biomarkers→ psychological factors→ lifestyle factors→ socioeconomic status** | | | | | |
| Mediated proportion | 39.64% (26.99%, 49.91%) | 13.25% (10.27%, 17.01%) | 30.60% (21.59%, 40.11%) | 12.54% (10.10%, 15.83%) | 3.97% (2.88%, 5.37%) |
| *P* | <0.001 | <0.001 | <0.001 | <0.001 | <0.001 |
| **childhood maltreatment→ biological biomarkers→ psychological factors→ socioeconomic status→ lifestyle factors** | | | | | |
| Mediated proportion | 39.64% (26.99%, 49.92%) | 13.25% (10.28%, 17.01%) | 30.60% (21.60%, 40.12%) | 4.89% (3.67%, 6.48%) | 11.62% (9.30%, 14.74%) |
| *P* | <0.001 | <0.001 | <0.001 | <0.001 | <0.001 |
| **childhood maltreatment→ biological biomarkers→ socioeconomic status→ psychological factors→ lifestyle factors** | | | | | |
| Mediated proportion | 40.42% (27.62%, 50.85%) | 13.49% (10.45%, 17.39%) | 6.32% (4.88%, 8.20%) | 27.92% (18.88%, 37.28%) | 11.85% (9.46%, 15.08%) |
| *P* | <0.001 | <0.001 | <0.001 | <0.001 | <0.001 |
| **childhood maltreatment→ biological biomarkers→ socioeconomic status→ lifestyle factors→ psychological factors** | | | | | |
| Mediated proportion | 42.34% (29.13%, 53.07%) | 14.09% (10.87%, 18.29%) | 6.49% (4.98%, 8.51%) | 14.04% (11.30%, 17.84%) | 23.04% (13.59%, 32.52%) |
| *P* | <0.001 | <0.001 | <0.001 | <0.001 | <0.001 |

Abbreviations: M1: M1-mediated effect; M2: M2-mediated effect; M3: M3-mediated effect; M4: M4-mediated effect

| Table S12 Estimated mediated proportion of selected mediators, by different temporal orders, for the association between childhood maltreatment and HPA-axis-related endocrine diseases, using sequential mediation analyses | | | | | |
| --- | --- | --- | --- | --- | --- |
|  | **Direct effect** | **Indirect effect** | | | |
|  |  | **M1** | **M2** | **M3** | **M4** |
| **childhood maltreatment→ socioeconomic status→ psychological factors → lifestyle factors →biological biomarkers** | | | | | |
| Mediated proportion | 27.81% (5.55%, 45.88%) | 38.00% (26.75%, 52.72%) | 24.55% (11.58%, 38.06%) | 9.05% (3.60%, 15.10%) | 0.58% (0.10%, 1.23%) |
| *P* | 0.020 | <0.001 | <0.001 | 0.005 | 0.031 |
| **childhood maltreatment→ socioeconomic status→ psychological factors → biological biomarkers→ lifestyle factors** | | | | | |
| Mediated proportion | 28.04% (5.60%, 46.26%) | 38.00% (26.65%, 52.85%) | 24.56% (11.45%, 38.17%) | 0.94% (0.39%, 1.72%) | 8.45% (3.12%, 14.29%) |
| *P* | 0.020 | <0.001 | 0.001 | 0.004 | 0.007 |
| **childhood maltreatment→ socioeconomic status→ lifestyle factors → biological biomarkers → psychological factors** | | | | | |
| Mediated proportion | 28.76% (5.80%, 47.40%) | 39.33% (27.63%, 54.92%) | 12.18% (5.72%, 19.75%) | 0.94% (0.30%, 1.80%) | 18.80% (5.13%, 32.02%) |
| *P* | 0.020 | <0.001 | 0.001 | 0.008 | 0.011 |
| **childhood maltreatment→ socioeconomic status→ lifestyle factors →psychological factors→ biological biomarkers** | | | | | |
| Mediated proportion | 28.56% (5.75%, 47.07%) | 39.21% (27.58%, 54.65%) | 12.14% (5.72%, 19.63%) | 19.49% (5.89%, 32.78%) | 0.60% (0.11%, 1.27%) |
| *P* | 0.020 | <0.001 | 0.001 | 0.008 | 0.031 |
| **childhood maltreatment→ socioeconomic status→ biological biomarkers →lifestyle factors→ psychological factors** | | | | | |
| Mediated proportion | 29.00% (5.85%, 47.75%) | 39.29% (27.51%, 55.03%) | 1.49% (0.75%, 2.52%) | 11.26% (5.00%, 18.54%) | 18.96% (5.19%, 32.35%) |
| *P* | 0.020 | <0.001 | <0.001 | 0.002 | 0.011 |
| **childhood maltreatment→ socioeconomic status→ biological biomarkers→ psychological factors → lifestyle factors** | | | | | |
| Mediated proportion | 28.31% (5.70%, 46.72%) | 38.19% (26.73%, 53.25%) | 1.45% (0.73%, 2.44%) | 23.53% (10.30%, 37.11%) | 8.53% (3.15%, 14.46%) |
| *P* | 0.020 | <0.001 | <0.001 | 0.002 | 0.007 |
| **childhood maltreatment→ psychological factors→ socioeconomic status → lifestyle factors →biological biomarkers** | | | | | |
| Mediated proportion | 24.15% (4.66%, 40.58%) | 44.96% (32.98%, 59.07%) | 22.53% (15.30%, 30.96%) | 7.86% (3.15%, 12.87%) | 0.51% (0.09%, 1.06%) |
| *P* | 0.019 | <0.001 | <0.001 | 0.005 | 0.032 |
| **childhood maltreatment→ psychological factors →socioeconomic status→ biological biomarkers → lifestyle factors** | | | | | |
| Mediated proportion | 24.35% (4.72%, 40.96%) | 44.97% (32.90%, 59.21%) | 22.52% (15.26%, 31.03%) | 0.82% (0.34%, 1.47%) | 7.34% (2.73%, 12.18%) |
| *P* | 0.020 | <0.001 | <0.001 | 0.004 | 0.007 |
| **childhood maltreatment→ psychological factors→ lifestyle factors→ socioeconomic status→ biological biomarkers** | | | | | |
| Mediated proportion | 24.00% (4.66%, 40.56%) | 43.51% (31.63%, 57.25%) | 17.24% (11.56%, 24.15%) | 14.75% (8.70%, 21.23%) | 0.50% (0.109%, 1.06%) |
| *P* | 0.020 | <0.001 | <0.001 | <0.001 | 0.031 |
| **childhood maltreatment→ psychological factors→ lifestyle factors→ biological biomarkers→ socioeconomic status** | | | | | |
| Mediated proportion | 24.12% (4.69%, 40.76%) | 43.47% (31.54%, 57.27%) | 17.23% (11.53%, 24.17%) | 0.76% (0.31%, 1.36%) | 14.42% (8.44%, 20.82%) |
| *P* | 0.020 | <0.001 | <0.001 | 0.005 | <0.001 |
| **childhood maltreatment→ psychological factors→ biological biomarkers→ lifestyle factors→ socioeconomic status** | | | | | |
| Mediated proportion | 24.29% (4.69%, 41.00%) | 43.51% (31.51%, 57.43%) | 1.43% (0.85%, 2.22%) | 16.25% (10.74%, 22.97%) | 14.52% (8.49%, 20.99%) |
| *P* | 0.020 | <0.001 | <0.001 | <0.001 | <0.001 |
| **childhood maltreatment→ psychological factors→ biological biomarkers→ socioeconomic status→ lifestyle factors** | | | | | |
| Mediated proportion | 24.52% (4.77%, 41.22%) | 44.90% (32.77%, 59.23%) | 1.29% (0.73%, 2.04%) | 21.91% (14.70%, 30.32%) | 7.39% (2.75%, 12.28%) |
| *P* | 0.020 | <0.001 | <0.001 | <0.001 | 0.007 |
| **childhood maltreatment→ lifestyle factors→ psychological factors→ socioeconomic status→ biological biomarkers** | | | | | |
| Mediated proportion | 25.42% (4.95%, 42.68%) | 24.02% (16.75%, 33.29%) | 34.40% (22.37%, 47.68%) | 15.63% (9.17%, 22.68%) | 0.53% (0.10%, 1.13%) |
| *P* | 0.020 | <0.001 | <0.001 | <0.001 | 0.031 |
| **childhood maltreatment→ lifestyle factors→ psychological factors→ biological biomarkers→ socioeconomic status** | | | | | |
| Mediated proportion | 25.55% (4.99%, 42.89%) | 24.00% (16.73%, 33.30%) | 34.37% (22.29%, 47.74%) | 0.80% (0.32%, 1.45%) | 15.28% (8.90%, 22.27%) |
| *P* | 0.020 | <0.001 | <0.001 | 0.005 | <0.001 |
| **childhood maltreatment→ lifestyle factors→ socioeconomic status→ psychological factors→ biological biomarkers** | | | | | |
| Mediated proportion | 27.90% (5.55%, 46.26%) | 26.97% (18.79%, 38.02%) | 25.50% (16.23%, 36.67%) | 19.04% (5.75%. 31.98%) | 0.59% (0.10%, 1.24%) |
| *P* | 0.020 | <0.001 | <0.001 | 0.008 | 0.031 |
| **childhood maltreatment→ lifestyle factors→ socioeconomic status→ biological biomarkers→ psychological factors** | | | | | |
| Mediated proportion | 28.09% (5.61%, 46.56%) | 27.05% (18.82%, 38.18%) | 25.57% (16.24%, 36.84%) | 0.91% (0.30%, 1.75%) | 18.36% (5.03%, 31.26%) |
| *P* | 0.020 | <0.001 | <0.001 | 0.008 | 0.011 |
| **childhood maltreatment→ lifestyle factors→ biological biomarkers→ psychological factors→ socioeconomic status** | | | | | |
| Mediated proportion | 28.24% (5.65%, 46.79%) | 27.01% (18.74%, 38.21%) | 1.41% (0.73%, 2.36%) | 24.89% (15.64%, 35.99%) | 18.46% (5.07%, 31.47%) |
| *P* | 0.020 | <0.001 | <0.001 | <0.001 | 0.011 |
| **childhood maltreatment→ lifestyle factors→ biological biomarkers→ socioeconomic status→ psychological factors** | | | | | |
| Mediated proportion | 25.73% (5.07%, 43.16%) | 24.11% (16.77%, 33.53%) | 1.35% (0.73%, 2.20%) | 33.43% (21.27%, 46.69%) | 15.38% (8.96%, 22.46%) |
| *P* | 0.020 | <0.001 | <0.001 | <0.001 | <0.001 |
| **childhood maltreatment→ biological biomarkers→ lifestyle factors→ socioeconomic status→ psychological factors** | | | | | |
| Mediated proportion | 25.91% (5.10%, 43.43%) | 2.40% (1.58%, 3.55%) | 22.52% (15.45%, 31.56%) | 33.67% (21.42%, 47.10%) | 15.50% (9.01%, 22.64%) |
| *P* | 0.020 | <0.001 | <0.001 | <0.001 | <0.001 |
| **childhood maltreatment→ biological biomarkers→ lifestyle factors→ psychological factors→ socioeconomic status** | | | | | |
| Mediated proportion | 28.45% (5.71%, 47.10%) | 2.58% (1.68%, 3.91%) | 25.29% (17.34%, 36.05%) | 25.08% (15.76%, 36.33%) | 18.60% (5.10%, 31.72%) |
| *P* | 0.020 | <0.001 | <0.001 | <0.001 | 0.011 |
| **childhood maltreatment→ biological biomarkers→ psychological factors→ lifestyle factors→ socioeconomic status** | | | | | |
| Mediated proportion | 24.57% (4.76%, 41.45%) | 2.27% (1.50%, 3.32%) | 42.04% (30.06%, 55.86%) | 16.44% (10.84%, 23.31%) | 14.69% (8.58%, 21.24%) |
| *P* | 0.020 | <0.001 | <0.001 | <0.001 | <0.001 |
| **childhood maltreatment→ biological biomarkers→ psychological factors→ socioeconomic status→ lifestyle factors** | | | | | |
| Mediated proportion | 24.77% (4.82%, 41.57%) | 2.11% (1.36%, 3.12%) | 43.53% (31.42%, 57.74%) | 22.13% (14.85%, 30.68%) | 7.46% (2.77%, 12.43%) |
| *P* | 0.020 | <0.001 | <0.001 | <0.001 | 0.007 |
| **childhood maltreatment→ biological biomarkers→ socioeconomic status→ psychological factors→ lifestyle factors** | | | | | |
| Mediated proportion | 28.48% (5.73%, 46.96%) | 2.31% (1.45%, 3.55%) | 36.95% (25.72%, 51.71%) | 23.68% (10.36%, 37.35%) | 8.58% (3.16%, 14.57%) |
| *P* | 0.020 | <0.001 | <0.001 | 0.002 | 0.007 |
| **childhood maltreatment→ biological biomarkers→ socioeconomic status→ lifestyle factors→ psychological factors** | | | | | |
| Mediated proportion | 29.19% (5.88%, 48.04%) | 2.38% (1.49%, 3.68%) | 38.03% (26.47%, 53.47%) | 11.33% (5.03%, 18.70%) | 19.08% (5.23%, 32.58%) |
| *P* | 0.020 | <0.001 | <0.001 | 0.002 | 0.007 |

Abbreviations: HPA: hypothalamic-pituitary-adrenal; M1: M1-mediated effect; M2: M2-mediated effect; M3: M3-mediated effect; M4: M4-mediated effect

Table S13 The competing risk of death in the relationship between childhood maltreatment and endocrine diseases

|  | **Number of death (%)/ Number of cases (%)** | | **Hazard Ratios of competing risk (95% CIs)*** |
| --- | --- | --- | --- |
|  | **childhood maltreatment experience <2** | **childhood maltreatment experience ≥2** |  |
| **Any endocrine diseases** |  |  |  |
|  | 1189(0.99)/15706(13.08) | 301(0.95)/5177(16.40) | 1.27(1.23-1.31) |
| **Individual endocrine diseases** |  |  |  |
| Hypothyroidism | 1493(1.24)/7076(5.89) | 382(1.21)/2378(7.54) | 1.25(1.20-1.31) |
| Hyperthyroidism | 1593(1.33)/1300(1.08) | 426(1.35)/429(1.36) | 1.21(1.09-1.35) |
| Thyroiditis | 1615(1.34)/318(0.26) | 433(1.37)/91(0.29) | 1.01(0.80-1.28) |
| Type 2 diabetes | 1387(1.15)/5423(4.52) | 359(1.14)/1924(6.10) | 1.42(1.35-1.49) |
| Hypoparathyroidism | 1621(1.35)/35(0.03) | 432(1.37)/14(0.04) | 1.38(0.73-2.61) |
| Hyperparathyroidism | 1607(1.34)/431(0.36) | 429(1.36)/143(0.45) | 1.26(1.04-1.52) |
| Hyperfunction of pituitary gland | 1608(1.34)/160(0.13) | 430(1.36)/47(0.15) | 1.11(0.81-1.54) |
| Hypofunction of pituitary gland | 1614(1.34)/208(0.17) | 430(1.36)/77(0.24) | 1.41(1.08-1.85) |
| Cushing syndrome | 1620(1.35)/31(0.03) | 431(1.37)/11(0.03) | 1.31(0.66-2.58) |
| Hyperaldosteronism | 1621(1.35)/36(0.03) | 433(1.37)/8(0.03) | 0.94(0.43-2.05) |
| Ovarian dysfunction | 1615(1.34)/398(0.33) | 433(1.37)/1.47(0.47) | 1.16(0.96-1.40) |
| Testicular dysfunction | 1618(1.35)/103(0.09) | 432(1.37)/21(0.07) | 0.88(0.55-1.40) |
| **Subtypes of endocrine diseases** |  |  |  |
| ***By involved gland*** |  |  |  |
| Endocrine diseases of thyroid gland | 1459(1.21)/8772(7.30) | 373(1.18)/2870(9.09) | 1.21(1.16-1.27) |
| Endocrine diseases of pancreatic gland | 1361(1.13)/6582(5.48) | 349(1.11)/2257(7.15) | 1.37(1.30-1.44) |
| Endocrine diseases of parathyroid gland | 1606(1.34)/460(0.38) | 428(1.36)/153(0.48) | 1.26(1.05-1.51) |
| Endocrine diseases of hypothalamic-pituitary gland | 1601(1.33)/347(0.29) | 428(1.36)/120(0.38) | 1.31(1.07-1.62) |
| Endocrine diseases of adrenal gland | 1600(1.34)/245(0.20) | 424(1.34)/101(0.32) | 1.56(1.23-1.96) |
| Endocrine diseases of genital gland | 1611(1.34)/501(0.42) | 432(1.37)/168(0.53) | 1.12(0.94-1.34) |
| ***By involved axis*** |  |  |  |
| Hypothalamic-pituitary-gonadal axis | 1591(1.32)/825(0.69) | 427(1.35)/283(0.90) | 1.21(1.06-1.39) |
| Hypothalamic-pituitary-thyroid axis | 1444(1.20)/9019(7.51) | 371(1.18)/2947(9.34) | 1.21(1.16-1.27) |
| Hypothalamic-pituitary-adrenal axis | 1580(1.32)/565(0.47) | 421(1.33)/207(0.66) | 1.39(1.18-1.63) |

* Competing risk models were adjusted for sex, birth year, ethnicity, country of birth, number of siblings, maternal smoking, being breastfed, and family history of diabetes

Table S14 Sensitivity analyses for the summarized mediated proportions of four hypothesized pathways from the sequential mediation analyses

| **Interested outcomes** | **Direct effect** | **Indirect effect** | | | | |
| --- | --- | --- | --- | --- | --- | --- |
|  |  | **Overall mediated effect** | **Suboptimal socioeconomic status** | **Psychological adversities** | **Unfavorable lifestyle** | **Biological alterations** |
| **Childhood maltreatment experience** ≥1 ***vs* <1** | | | | | | |
| **Any endocrine diseases** | 30.89%~34.31% | 65.69%~69.11% | 3.81%~7.20% | 38.38%~48.74% | 9.44%~19.44% | 1.38%~8.56% |
| **Type 2 diabetes** | 39.37%~44.83% | 55.17%~60.63% | 3.23%~6.23/% | 21.84%~35.66% | 10.40%~24.23% | 3.11%~14.13% |
| **HPA-axis-related endocrine diseases** | 23.04%~28.53% | 71.47%~76.96% | 13.05%~38.28% | 19.71%~47.80% | 7.32%~27.51% | 0.45%~2.43% |
| **Childhood maltreatment experience** ≥**2 *vs* =0** | | | | | | |
| **Any endocrine diseases** | 41.94%~45.91% | 54.09%~58.06% | 3.41%~6.07% | 30.75%~40.45% | 8.52%~16.98% | 0.90%~6.83% |
| **Type 2 diabetes** | 39.39%~45.07% | 54.93%~60.61% | 3.68%~6.90% | 20.25%~34.79% | 10.84%~25.30% | 2.86%~14.07% |
| **HPA-axis-related endocrine diseases** | 22.96%~28.84% | 71.16%~77.04% | 14.16%~39.75% | 18.24%~ 46.63% | 6.93%~27.52% | 0.48%~2.55% |
| **Multiple imputation to deal with missing values** | | | | | | |
| **Any endocrine diseases** | 39.57%~42.45% | 57.55%~60.43% | 3.49%~6.21% | 36.64%~44.53% | 7.79%~14.80% | 0.70%~5.08% |
| **Type 2 diabetes** | 33.84%~38.19% | 61.81%~66.16% | 4.29%~7.79% | 27.10%~40.01% | 11.59%~25.61% | 2.39%~12.14% |
| **HPA-axis-related endocrine diseases** | 23.59%~28.60% | 71.40%~76.41% | 14.10%~39.07% | 15.03%~ 45.97% | 7.24%~27.32% | 0.44%~2.11% |

HPA: hypothalamic-pituitary-adrenal;
