# Supplementary material for: Childhood maltreatment and risk of endocrine diseases: an exploration of mediating pathways using sequential mediation analysis
Source: BMC Med. 2024 Feb 8;22:59. doi: 10.1186/s12916-024-03271-9 (PMC10854183; doi:10.1186/s12916-024-03271-9)
Supplement: Supplementary file 2 — Additional file 2. Supplementary method. [file 12916_2024_3271_MOESM2_ESM.docx]

**_Childhood maltreatment and risk of endocrine diseases: an exploration of mediating pathways using sequential mediation analysis_**

**Additional file 2**

Shu Wen; Jianwei Zhu; Xin Han; Yuchen Li; Haowen Liu; Huazhen Yang; Can Hou; Shishi Xu; Junren Wang; Yao Hu; Yuanyuan Qu; Di Liu; Thor Aspelund; Fang Fang; Unnur A Valdimarsdóttir; Huan Song

**Additional file 2**

**Contents**

[Supplementary method 3](#_Toc156565498)

Supplementary method

**Mediators**

In the current study, we extracted candidate mediators in four categories: socioeconomic status (SES), psychological factors, lifestyle factors and biological factors, the detailed questions for these mediators in UK Biobank were available in Additional file 1: Table S2.

***Socioeconomic status***

Townsend Deprivation Index (TDI) was derived from preceding national census data and based on the area-based proxy measures for unemployment, non-car ownership, non-home ownership and household overcrowding[39]. Score 0 indicated the mean value for an area and the higher score implied the more deprived the participants were[39]. Household income was classified as <£18,000, £18,000-30,999, £31,000-51,999, £52,000-100,000 and >£100,000 annually. Education level was classified as university qualifications (college or university degree) and non-university qualifications. As UK Biobank regarded as a community-based cohort identifying the causes of diseases of middle and old age, we classified education level as employed (including those in paid employment or self-employed, or retired) and other in the current study.

***Psychological factors***

For mental health assessment, we defined a positive answer (yes) to either of the two questions (i.e., “Have you ever seen a general practitioner (GP) for nerves, anxiety, tension or depression?” and "Have you ever seen a psychiatrist for nerves, anxiety, tension or depression?") as self-rated mental problem[6]. Moreover, social support was deemed as field on psychological factors, including ability to confide in others, frequency of visitors and leisure/ social activities. The first two variables were recoded as dichotomous groups (i.e., “less than weekly” versus “weekly or more”), with leisure/ social activities recoded as “no place” (i.e., no place once a week or more often) and “at least one place” (i.e., at least one place once a week or more often)[40].

***Lifestyle factors***

Physical activity (PA) was measured by total metabolic equivalent task (MET) minutes per week, grouped as low PA (<600 MET-mins/week), moderate PA (600 to < 3000 MET-mins/week), and high PA (≥3000 MET-mins/week) according to previous study[41]. Smoking status was grouped as “never” and “ever” (i.e., previous or current smoking). The frequency of alcohol intake was grouped as “never/ special occasion” and “drinking” (i.e., both “less than 3 times weekly” and “at least 3 times weekly”). In accordance with previous study, five sleep factors (i.e., chronotype, duration, insomnia, snoring and excessive daytime sleepiness) were combined to generate a health sleep score, which ranged from 0 to 5. The sleep pattern was grouped as “health sleep pattern” (i.e., sleep score ≥ 4), “intermediate sleep pattern” and “poor sleep pattern” (i.e., sleep score ≤ 1)[42]. Similarly, a diet score was created with the sum of key food groups intake based on previous study, including fruit and vegetables group, whole grains/cereal group, processed meat group and red meat group, each group was scored from 0 to 0.5[43]. Total diet score ranged from 0 to 2, with higher score representing healthier diet habit. Body mass index (BMI) was calculated as weight (kg) divided by height (m) squared and was used as continuous variable. Resting systolic pressure and diastolic pressure were measured twice and the mean value of the two measuring results was used in the study.

***Biological biomarkers***

We screened C-reactive protein for inflammation, total protein, Ca and Vitamin D for nutrition, cholesterol, lipoprotein A, high density lipoprotein, low density lipoprotein, apolipoprotein A, apolipoprotein B and triglycerides for lipids, glucose and glycosylated hemoglobin (HbA1c) for glucose as mediators in biological category. These variables were analyzed as continuous data.

**Mediation analysis**

***Simple mediation analysis***

The decomposition that the total effect (TE) was decomposed into direct effect (DE) and indirect effect (IE) yielded valid results under a set of structural and parametric assumptions as follows: (1) conditional on covariates, also called confounders, there was no unmeasured confounding for the exposure-outcome relationship; (2) conditional on confounders, there is no unmeasured confounding for the mediator-outcome relationship; (3) conditional on confounders, there is no unmeasured confounding of the exposure-mediator relationship; (4) no effects of exposure that confounded the mediator-outcome relationship[46]. G-formula approach able to deal with mediator-outcome confounders (i.e., post-confounders) affected by the exposure was selected, yet post-confounders not taken into consideration in our study[47].

***Sequential mediation analysis***

The method for sequential mediation models was extended from Huang et al[30], which was generalized to incorporate survival outcome and multiple mediators. Taking advantage of the conjugacy property of normal distributions, path-specific effects could be approximated based on resample-based method[48,49] and counterfactual notions when no interaction and no time-varying confounders were assumed[50]. Hence, a series of no unmeasured confounding assumptions needed to be made to identify the effects of these pathways: (1) no unmeasured confounding for the joint effect of the four mediators $(M1, M2, M3, M4)$ on outcome, conditional on exposure; (2) no unmeasured confounding for the effect of exposure $S$ to outcome; (3) no unmeasured confounding for the effect of exposure $S$ on M1; (4) no unmeasured confounding for the joint effect of exposure and M1 $(S, M1)$on M2; (5) no unmeasured confounding for the joint effect of exposure, M1 and M2 $(S, M1, M2)$ on M3; (6) no unmeasured confounding for the joint effect of exposure, M1, M2 and M3 $(S, M1, M2, M3)$ on M4; (7) no alternative path from the exposure to the M1 and outcome through an unknown common mediator; (8) no alternative path from exposure to the M2 and outcome through a common mediator other than M1; (9) no alternative path from exposure to the M3 and outcome through a common mediator other than M1 and M2; (10) no alternative path from exposure to the M4 and outcome through a common mediator other than M1, M2 and M3; (11) no alternative path sequentially from the exposure to M1, M2, M3 and M4 through an unknown common mediator[30]. Besides, standard assumptions of positively and consistency were required[51]. As mentioned before, variances and confidence intervals of the model could be calculated using resampling method[49]. The detailed explanation was provided as follows:

Let $s$ be the exposure (i.e., childhood maltreatment) and let Y be the defined outcome (i.e., any endocrine diseases, type 2 diabetes and HPA-axis-related endocrine diseases). Let $M1$, $M2$, $M3$, $M4$ be the four mediators in order. We define the following nested counterfactuals. Let

$$M1(s')$$

be the counterfactual outcome of $M1$ under the **counterfactual 1** defined as: for each subject, $M1$ is set to the value it would have if $s$ is set to $s'$. Let

$$M2\left( s^{''}, M1\left( s^{'} \right) \right)$$

be the counterfactual outcome of $M2$ under the **counterfactual 2** defined as: for each subject, $M2$ is set to the value it would have if $s$ is set $s''$ and **counterfactual 1** is simultaneously performed. Let

$$M3 \left( s^{'''},M1 \left( s^{'} \right),M2 \left( s^{''},M1 \left( s^{'} \right) \right) \right)$$

be the counterfactual outcome of $M3$ under the **counterfactual 3** defined as: for each subject, $M3$ is set to the value it would have if $s$ is set $s'''$ and counterfactuals 1 and 2 are simultaneously performed. Let

$$M4\left( s^{''''},M1\left( s^{'} \right),M2\left( s^{''},M3\left( s^{'} \right) \right) , M4\left( s^{'''},M1\left( s' \right),M2\left( s'',M1(s' \right) \right) \right)$$

be the counterfactual outcome of $M4$ under the **counterfactual 4** defined as: for each subject, $M4$ is set to the value it would have if $s$ is set $s''''$ and counterfactuals 1,2 and 3 are simultaneously performed. Finally let

$$Y\left( s,M1 \left( s^{'} \right),M2 \left( s^{''},M1 \left( s^{'} \right) \right),M3 \left( s^{'''},M1 \left( s^{'} \right),M2 \left( s^{''},M1 \left( s^{'} \right) \right) \right),M4 \left( s^{''''},M1 \left( s^{'} \right),M2 \left( s^{''},M1 \left( s^{'} \right) \right) ,M3 \left( s^{'''},M1 \left( s^{'} \right),M2 \left( s^{''},M1 \left( s^{'} \right) \right) \right) \right) \right)$$

be the counterfactual value of $Y$ under the **counterfactual 4** defined as: for each subject, $Y$ is set to the value it would have if $s$ were set to $s$ and counterfactuals 1, 2, and 3 are simultaneously performed.

As reported by Huang et al[30], we proposed the following five models for the four mediators $M1$, $M2$, $M3$, $M4$, and the transformed survival time $H(T)$:

$M1i =\delta_{X}^{T}Xi + \delta_{s}Si +\epsilon_{M1i}$, where $\epsilon_{M1i} \sim N (0, \sigma_{M1}^{2})$

$M2i = \alpha_{X}^{T}Xi+\alpha_{s}Si +\alpha_{M1}M1i +\epsilon_{M2i}$, where$\epsilon_{M2i} \sim N (0, \sigma_{M2}^{2})$

$M3i = \beta_{X}^{T}Xi+\beta_{s}Si +\beta_{M1}M1i +{\beta_{M2}M2i+ \epsilon}_{M3i}$, where$\epsilon_{M3i} \sim N (0, \sigma_{M3}^{2})$

$M4i = \theta_{X}^{T}Xi+\theta_{s}Si +\theta_{M1}M1i+\theta_{M2}M2i+\theta_{M3}M3i+\epsilon_{M3i}$, where$\epsilon_{M3i} \sim N \left( 0, \sigma_{M3}^{2} \right)$

$$log\lambda i = log\lambda_{0}\left( t \right)+\gamma_{X}^{T}X_{i}^{*}+\gamma_{s}Si +\gamma_{M1}M1i+\gamma_{M2}M2i+\gamma_{M3}M3i+\gamma_{M4}M4i$$

$$= log\lambda_{0}\left( t \right)+\gamma_{X}^{T}X_{i}^{*}+\gamma_{s}Si + W_{\gamma i}$$

where $W_{\gamma i}= \gamma_{M1}M1i+\gamma_{M2}M2i+\gamma_{M3}M3i+\gamma_{M4}M4i$, $W_{\gamma i}$ is a function of $s'$, $s''$, $s'''$ and $s''''$ following a normal distribution $G_{W_{\gamma i}}$: $W_{\gamma i}(s^{'}, s^{''}, s^{'''}, s'''' ) \sim N (\mu_{W_{\gamma i}}, \sigma_{W_{\gamma}}^{2})$, which would hold under the assumptions of no unmeasured confounding.

The estimated DE following causal effects.

$$DE= \Delta{Cox}_{(S\to Y)}= log\lambda(T (s1, M1(s0), M2(s0, M1(s0)), M3(s0, M1(s0), M2(s0, M1(s0))), M4(s0, M1(s0), M2(s0, M1(s0)), M3(s0, M1(s0), M2(s0, M1(s0))))) | X) - log\lambda(T \left( s0, M1\left( s0 \right), M2\left( s0, M1\left( s0 \right) \right), M3\left( s0, M1\left( s0 \right), M2\left( s0, M1\left( s0 \right) \right) \right), M4\left( s0, M1\left( s0 \right), M2\left( s0, M1\left( s0 \right) \right), M3\left( s0, M1\left( s0 \right), M2\left( s0, M1\left( s0 \right) \right) \right) \right) \right)\left| X \right)\approx\gamma_{s}(s1-s0)$$

The $M4$ mediated path-specific effect:

$$\Delta{Cox}_{\left( S\to M4\to Y \right)} =log\lambda(T (s1, M1(s0), M2(s0, M1(s0)), M3(s0, M1(s0), M2(s0, M1(s0))), M4(s1, M1(s0), M2(s0, M1(s0)), M3(s0, M1(s0), M2(s0, M1(s0))))) | X) - log\lambda(T \left( s1, M1\left( s0 \right), M2\left( s0, M1\left( s0 \right) \right), M3\left( s0, M1\left( s0 \right), M2\left( s0, M1\left( s0 \right) \right) \right), M4\left( s0, M1\left( s0 \right), M2\left( s0, M1\left( s0 \right) \right), M3\left( s0, M1\left( s0 \right), M2\left( s0, M1\left( s0 \right) \right) \right) \right) \right)\left| X \right) \approx\gamma_{M4}\theta_{s}(s1-s0)$$

The $M3$ mediated path-specific effect:

$$\Delta{Cox}_{\left( S\to M3\to Y \right)}= log \lambda(T (s1, M1(s0), M2(s0, M1(s0)), M3(s1, M1(s0), M2(s0, M1(s0))), M4(s1, M1(s0), M2(s0, M1(s0)), M3(s1, M1(s0), M2(s0, M1(s0))))) | X) - log \lambda(T \left( s1, M1\left( s0 \right), M2\left( s0, M1\left( s0 \right) \right), M3\left( s0, M1\left( s0 \right), M2\left( s0, M1\left( s0 \right) \right) \right), M4\left( s1, M1\left( s0 \right), M2\left( s0, M1\left( s0 \right) \right), M3\left( s0, M1\left( s0 \right), M2\left( s0, M1\left( s0 \right) \right) \right) \right) \right)\left| X \right)\approx(\gamma_{M3}+\gamma_{M4}\theta_{M3}) \beta_{s} (s1-s0)$$

The $M2$ mediated path-specific effect:

$$\Delta{Cox}_{\left( S\to M2\to Y \right)}=log \lambda(T (s1,M1(s0),M2(s1,M1(s0)),M3(s1,M1(s0),M2(s1,M1(s0))),M4(s1,M1(s0),M2(s1,M1(s0)),M3(s1,M1(s0),M2(s1,M1(s0))))) | X) - log \lambda(T \left( s1,M1\left( s0 \right),M2\left( s0,M1\left( s0 \right) \right),M3\left( s1,M1\left( s0 \right),M2\left( s0,M1\left( s0 \right) \right) \right),M4\left( s1,M1\left( s0 \right),M2\left( s0,M1\left( s0 \right) \right),M3\left( s1,M1\left( s0 \right),M2\left( s0,M1\left( s0 \right) \right) \right) \right) \right)\left| X \right) \approx(\gamma_{M2}+\gamma_{M3}\beta_{M2}+\gamma_{M4}\theta_{M3}\beta_{M2}+\gamma_{M4}\theta_{M2})\alpha_{s} (s1-s0)$$

The $M1$ mediated path-specific effect:

$$\Delta{Cox}_{\left( S\to M1\to Y \right)}=log \lambda(T (s1, M1(s1), M2(s1, M1(s1)), M3(s1, M1(s1), M2(s1, M1(s1))), M4(s1, M1(s1), M2(s1, M1(s1)), M3(s1, M1(s1), M2(s1, M1(s1))))) | X) - log \lambda(T (s1, M1(s0), M2(s1, M1(s0)), M3(s1, M1(s0), M2(s1, M1(s0))), M4(s1, M1(s0), M2(s1, M1(s0)), M3(s1, M1(s0), M2(s1, M1(s0))))) | X) \approx(\gamma_{M1}+\gamma_{M2}\alpha_{M1} +\gamma_{M3}\beta_{M2}\alpha_{M1}+ \gamma_{M3}\beta_{M1}+\gamma_{M4}\theta_{M3}\beta_{M2}\alpha_{M1}+ \gamma_{M4}\theta_{M3}\beta_{M1} +\gamma_{M4}\theta_{M2}\alpha_{M1}+\gamma_{M4}\theta_{M1}) \delta_{s} (s1-s0)$$

From these definitions of causal effects, we have that

$$IE= \Delta{Cox}_{\left( S\to M1\to Y \right)} +\Delta{Cox}_{\left( S\to M2\to Y \right)} +\Delta{Cox}_{\left( S\to M3\to Y \right)} + \Delta{Cox}_{\left( S\to M4\to Y \right)}$$

And TE:

$$TE= DE+IE$$

$X$: covariates

**Sensitivity analysis**

In the sensitivity analyses, we firstly re-estimated those mediation proportions using individuals without any childhood maltreatment as reference (i.e., childhood maltreatment ≥2 *vs* 0). Secondly, missing values were dealt with multiple imputation, which imputed missing values by drawing from the posterior predictive distribution of the unobserved data given by the observed data. We created 50 imputed datasets with multiple imputation method (“MICE” package), in which univariate imputation models were specified for each mediator with missing data.
